# Functional Complete Revascularization as Determined by an Optimized Scoring System After Revascularization: A Post Hoc Analysis from Multi‐Center PANDA III Trial

**DOI:** 10.1002/advs.202415961

**Published:** 2025-02-18

**Authors:** Rui Zhang, Shaoyu Wu, Qianqian Liu, Changdong Guan, Hao‐Yu Wang, Sheng Yuan, Lihua Xie, Yunfei Huang, Zheng Qiao, Weida Liu, Rui Fu, Lei Feng, Chenggang Zhu, Lei Song, Dong Yin, Kefei Dou

**Affiliations:** ^1^ Cardiometabolic Medicine Center National Clinical Research Center for Cardiovascular Diseases Fuwai Hospital National Center for Cardiovascular Diseases Chinese Academy of Medical Sciences and Peking Union Medical College Beijing 100037 China; ^2^ Department of Cardiology National Clinical Research Center for Cardiovascular Diseases Fuwai Hospital National Center for Cardiovascular Diseases Chinese Academy of Medical Sciences and Peking Union Medical College Beijing 100037 China; ^3^ State Key Laboratory of Cardiovascular Disease Beijing 102300 China; ^4^ Department of Cardiology Guangdong Provincial People's Hospital (Guangdong Academy of Medical Sciences) Southern Medical University Guangzhou 510080 China; ^5^ Catheterization Laboratories National Clinical Research Center for Cardiovascular Diseases Fuwai Hospital National Center for Cardiovascular Diseases Chinese Academy of Medical Sciences and Peking Union Medical College Beijing 100037 China; ^6^ State Key Laboratory for Complex Severe, and Rare Diseases Peking Union Medical College Hospital Chinese Academy of Medical Sciences Beijing 100730 China; ^7^ National Clinical Research Center for Cardiovascular Diseases Fuwai Hospital Chinese Academy of Medical Sciences Shenzhen 518038 China

**Keywords:** coronary artery disease, functional complete revascularization, percutaneous coronary intervention, quantitative flow ratio, residual functional SYNTAX score

## Abstract

Functional complete revascularization (CR) after percutaneous coronary intervention (PCI) as determined by classic residual functional SYNTAX score (c‐rFSS) has been associated with improved prognosis. In this study, the c‐rFSS algorithm is optimized for a novel modified rFSS (m‐rFSS) and prognostic implications of this novel scoring is determined. The m‐rFSS algorithm is updated for 2 clinical scenarios, i.e., 1) lesions with suboptimal functional results, and 2) angiographic diameter stenosis <50% but functionally significant stenoses, which are not scored by c‐rFSS. The major outcome is a 2‐year major adverse cardiac event (MACE). A total of 1,555 patients analyzable for both c‐rFSS and m‐rFSS are included. After calculating m‐rFSS, 12.0% (187/1,555) of patients with c‐rFSS‐based functional CR (c‐rFSS = 0) are reclassified as having m‐rFSS‐based incomplete revascularization (IR, m‐rFSS>0); thus, 377 (21.7%) patients have c‐rFSS‐based functional IR whereas 524 (33.7%) has m‐rFSS‐based IR. Patients with m‐rFSS‐based functional IR (m‐rFSS>0) show a significantly higher risk for major MACE outcome (20.8% vs 5.9%; adjusted hazard ratio 3.32, 95% confidence interval: 2.34–4.71) than patients with functional CR (m‐rFSS = 0). The m‐rFSS is more predictive of 2‐year MACE than c‐rFSS (difference in C‐index 0.07, *p* < 0.001). In this study, we optimized the classic scoring algorithm to develop a novel scoring system (m‐rFSS), and revascularization completeness determined by m‐rFSS is markedly associated with a 2‐year prognosis.

## Graphical abstract

The modified residual functional SYNTAX score (m‐rFSS) algorithm was updated for 2 clinical scenarios: 1) lesions with suboptimal functional results, and 2) angiographic diameter stenosis <50% but functionally significant stenoses, which were not scored by classic rFSS or rSS. CR = complete revascularization; IR = incomplete revascularization; PCI = percutaneous coronary intervention; QFR = quantitative flow ratio.

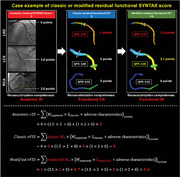

## 1 Introduction

Coronary physiology‐guided percutaneous coronary intervention (PCI) has improved long‐term prognosis in large clinical studies.^[^ ^1^, ^2^, ^3^, ^4^, ^5^, ^6^ ^]^ Multi‐scenario application of intracoronary physiology (e.g., fractional flow reserve [FFR] and quantitative flow ratio [QFR]) has been recommended by the latest international consensus and guidelines,^[^ ^7^, ^8^, ^9^ ^]^ which includes postprocedural assessment of PCI completeness. In fact, the definition of complete revascularization (CR) after PCI has moved from the concept of “anatomic” to “functional” CR in recent decades.^[^ ^10^ ^]^ The residual SYNTAX score (rSS) is a comprehensive angiographic scoring system that is used to objectively quantify the anatomic complexity of residual disease burden after PCI.^[^ ^11^ ^]^ The postprocedural residual functional SYNTAX score (rFSS) integrates the coronary anatomy (i.e., rSS) and physiology (i.e., post‐PCI coronary physiology) by only summing the individual scores of angiographically qualifying lesions that are also hemodynamically flow‐limiting and is applied to define whether functional CR has been achieved. This metric has been proven to have superior prognostic value to rSS alone.^[^ ^12^, ^13^ ^]^

However, the classic rFSS (hereafter called c‐rFSS) can only downgrade severity and may underestimate prognostic risk due to its inherent limitations. The c‐rFSS only takes into account lesions that are angiographically obstructive (diameter stenosis [DS] ≥50%). Since assessment mismatch between coronary physiology and anatomy is not uncommon,^[^ ^14^, ^15^, ^16^, ^17^, ^18^ ^]^ c‐rFSS may undershoot the impact of angiographic DS <50% but functionally significant residual stenoses, while such lesions are actually associated with adverse prognosis. Moreover, c‐rFSS sums only lesions with residual functional ischemia (post‐PCI FFR or QFR ≤0.80). Considering that growing evidence suggests that post‐PCI suboptimal functional results (e.g., post‐PCI FFR or QFR ≤0.90) are prognostically adverse despite the absence of hemodynamically flow‐limiting (post‐PCI FFR or QFR >0.80),^[^ ^18^, ^19^, ^20^, ^21^ ^]^ the prognostic value of such residual lesions is overlooked.

To optimize the algorithms used to determine rFSS in light of the aforementioned two scenarios, we derived the modified rFSS (m‐rFSS). In the present study, we analyzed the post‐PCI QFR, anatomic rSS, c‐rFSS, and m‐rFSS of patients enrolled in the all‐comers randomized PANDA III (Comparison of BuMA eG Based Biodegradable Polymer Stent With EXCEL Biodegradable Polymer Sirolimus‐eluting Stent in “Real‐World” Practice) (NCT02017275),^[^ ^22^ ^]^ and investigated the prognostic implication of m‐rFSS and its derived assessment of functional PCI completeness in comparison with those of c‐rFSS and anatomic rSS.

## 2 Experimental Section

### 2.1 Panda III Trial and the Present Study

The original PANDA III trial was a multicenter trial in which 2348 patients were randomized to two biodegradable polymer‐based sirolimus‐eluting stents with differing elution and absorption kinetics. The 1‐year and 2‐year rates of major adverse cardiac events (MACE) were similar with both stent types.^[^ ^22^, ^23^ ^]^ Data from 2 arms were thus pooled for the present analysis. The present study was a *post hoc* analysis from the PANDA III trial in which pre‐ and post‐PCI QFR analyses as well as calculations of functional SYNTAX score were retrospectively performed. The PANDA III trial and the present study were approved by an institutional review committee and performed in accordance with the Declaration of Helsinki. All patients provided written informed consent.

### 2.2 QFR Analysis and Angiographic Parameters

The QFR is a novel angiography‐based computational physiological index that has been validated as having good reproducibility and diagnostic accuracy compared with FFR as the reference standard.^[^ ^24^ ^]^ The latest randomized trial (NCT03656848) demonstrated that QFR‐guided PCI improved 1‐ and 2‐year clinical outcomes compared with standard angiography‐guided PCI.^[^ ^6^, ^25^ ^]^ In this study, the pre‐and post‐PCI QFR analyses were retrospectively performed in 2 independent core laboratories (CCRF, Beijing, China; Interventional Cardiovascular Imaging Core Laboratory, National Center for Cardiovascular Diseases, Beijing, China). Overall, off‐line analysis of QFR was performed using the AngioPlus system (Pulse Medical Imaging Technology, Shanghai, China) based on two angiograms with projection angles ≥25° from pre‐ and post‐PCI coronary angiograms. Baseline and post‐PCI QFR values were obtained following a standard procedure.^[^ ^6^, ^24^ ^]^

Quantitative coronary angiography (QCA) characteristics, including the reference vessel diameter (RVD), minimal lumen diameter (MLD), DS, and lesion length, were analyzed in a blinded way at an independent core laboratory using well‐validated software (QAngio version 7.3; Medis Medical Imaging Systems, Leiden, the Netherlands).

### 2.3 Anatomic and Classic Residual Functional SYNTAX Score

**Figure 1:**
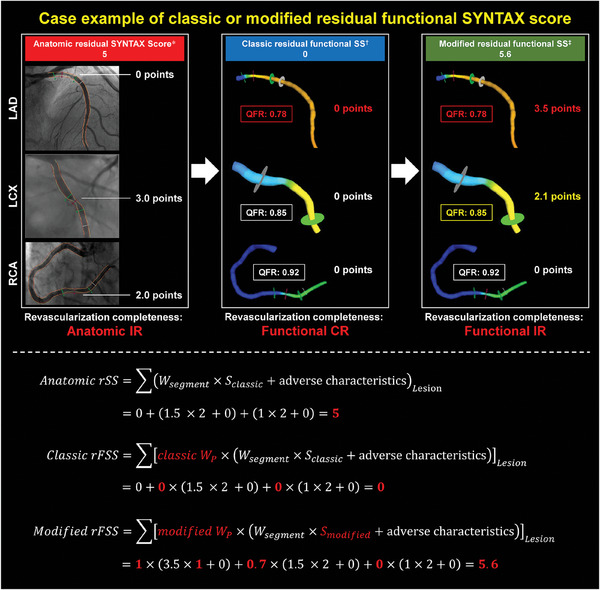
Representative Case of Different Scoring Systems. *Anatomic residual SYNTAX score (rSS): *W_segment_* is for segment weighing, *S_classic_* is for classic multiplication factor for severity in which a multiplication factor of 2 is used for non‐occlusive lesions and 5 for occlusive lesions. *Adverse* *characteristics* refer to the scores for lesion adverse characteristics. ^†^Classic functional residual SYNATX score (c‐rFSS): *classic* *W_P_* refer to classic physiology weighting with a multiplication factor of 1 for lesions with post‐PCI QFR ≤0.80 and 0 for QFR >0.80; ^‡^Modified functional residual SYNATX score (m‐rFSS): *modified* *W_P_* refer to modified physiology weighting, with a multiplication factor of 1 for lesions with post‐PCI vessel QFR ≤0.80, 0.7 for QFR between 0.80 to 0.90, and 0 for QFR >0.90; *S_modified_* is for modified multiplication factor for severity in which a multiplication factor of 5 is used for occlusive lesions, 2 for non‐occlusive lesions, and 1 for nonobstructive plaque. A patient yielded an anatomic rSS of 5, with a score of 0 for the left anterior descending (LAD) due to the diameter stenosis in LAD of less than 50% by visual assessment, and this patient was therefore stratified into the anatomic incomplete revascularization group. In the patient, the c‐rFSS was as low as 0 because the functional assessment using QFR revealed that the lesions in the left circumflex artery (LCX) and right coronary artery (RCA) were not significant (post‐PCI QFR >0.80) whereas the LAD had an anatomic rSS of 0 despite the presence of functional ischemia (post‐PCI QFR ≤0.80). For m‐rFSS, the score was 5.6 points because optimized scoring rules were applied in case of suboptimal functional result (post‐PCI QFR: 0.80‐0.90) (LCX, 2.1 points) and mismatch in anatomy and physiology (LAD, 3.5 points). CR = complete revascularization; IR = incomplete revascularization; LAD = left anterior descending; LCX = left circumflex artery; SS = SYNTAX score; PCI = percutaneous coronary intervention; QFR = quantitative flow ratio.

The baseline SYNTAX score, post‐PCI rSS, c‐rFSS, and m‐rFSS were calculated by an independent core laboratory. From the baseline and postprocedural angiograms, the baseline SYNTAX score and rSS were determined using an online calculator using a specific scoring algorithm.^[^ ^11^, ^26^ ^]^ Specifically, each coronary lesion with visual DS ≥50% in vessels ≥1.5 mm was separately scored, and the scores were summed to yield the overall anatomic SYNTAX score. The calculation of anatomic rSS was mainly based on the following lesion parameters: segment weighing, the multiplication factor for severity, and adverse characteristic scoring.^[^ ^26^ ^]^ The formula can be simplified as follows (**Figure** **1**):

(1) $$rSS=\sum {\left(W_{segment}\times S_{classic}+\mathrm{adverse}\;\mathrm{characteristics}\right)}_{Lesion}$$

*W_segment_* means segment weighing and was determined by coronary dominance and lesion location. *S_classic_* is for classic multiplication factor for severity in which a multiplication factor of 2 was used for nonocclusive lesions (DS 50–99%) and 5 for occlusive lesions (DS 100%), reflecting the difficulty of percutaneous treatment; and *Adverse* *characteristics* refers to the scores for adverse lesion characteristics (e.g., total occlusion, bifurcation, tortuosity, length, and calcification).

The c‐rFSS was calculated by summing only the individual rSS of lesions in vessels with hemodynamically flow‐limiting (post‐PCI FFR or QFR ≤0.80).^[^ ^12^, ^13^ ^]^ The formula for c‐rFSS can be simplified as follows (Figure 1):

(2) $$ClassicrFSS=\sum {\left[classicW_{P}\times \left(W_{segment}\times S_{classic}+\mathrm{adverse}\;\mathrm{characteristics}\right)\right]}_{Lesion}$$

*classic* *W_P_* refers to the classic physiology weighting, with a multiplication factor of 1 for lesions with post‐PCI vessel FFR or QFR ≤0.80 and 0 for lesions with vessel FFR or QFR >0.80.

### 2.4 Modified Residual Functional SYNTAX Score

As with rSS and c‐rFSS, m‐rFSS takes into account all vessels regardless of stenting. Compared with c‐rFSS, m‐rFSS has been optimized for two aspects: 1) given that the increasing emphasis on the prognostic impact of suboptimal functional results for vessels treated with PCI,^[^ ^18^, ^19^, ^20^, ^21^ ^]^ an extra physiology weighting of 0.7 was assigned to lesion with QFR range from 0.80 to 0.90 in vessels after stenting (details of the weighting determination was shown in the Supporting Information); and 2) considering the impact of lesions with angiographic DS <50% but functionally significant stenoses were underestimated,^[^ ^14^, ^15^, ^16^, ^17^, ^18^ ^]^ *S_classic_* updated to *S_modified_* in which a multiplication factor of 1 was extra used for nonobstructive coronary plaque (DS <50%). The formula for m‐rFSS is as follows (Figure 1):

(3) $$\begin{array}{cc} Modified\;rFSS & =\sum [modifiedW_{P}\times (W_{segment}\times S_{modified}\\ & \;{+\mathrm{adverse}\;\mathrm{characteristics})]}_{Lesion} \end{array}$$

*modified* *W_P_* refers to the modified physiology weighting, with a multiplication factor of 1 for lesions with post‐PCI QFR ≤0.80, 0.7 for QFR between 0.80 and 0.90, and 0 for QFR >0.90. The details of the determination of *modified* *W_P_* are described in the Supporting Information. *S_modified_* is for modified multiplication factor for severity in which a multiplication factor of 5 was used for occlusive lesions, 2 for non‐occlusive lesions, and 1 for nonobstructive plaque (DS <50%).

Anatomic CR was defined as anatomic rSS = 0, and functional CR was defined as a c‐rFSS or m‐rFSS = 0. The key differences among rSS, c‐rFSS, and m‐rFSS are shown in Table S1 (Supporting Information).

### 2.5 Clinical Outcomes and Follow‐Up

The major outcome of the present study was the two‐year rate of MACE, defined as the composite of all‐cause death, all MI, or any ischemia‐driven revascularization. Secondary outcomes included MACE excluding periprocedural myocardial infarction (MI), the individual components of MACE, and stent thrombosis. All definitions of clinical endpoints were identical to the PANDA III trial.^[^ ^22^ ^]^ All adverse events were adjudicated by a clinical events committee.

### 2.6 Statistical Analysis

Continuous variables were expressed as mean ± SD or median (interquartile range [IQR]) and were compared using the Student's *t*‐test or the Mann‐Whitney U test as appropriate. Categorical variables were presented as count and percentage and were compared using the chi‐square test or Fisher's exact test as appropriate. The 1.6% of the creatinine clearance and 4.1% of the left ventricular ejection fraction were missing data and handled by single imputation with a median. Correlations among anatomic rSS, c‐rFSS, and m‐rFSS were tested using the Spearman correlation coefficient (r). The cumulative incidence of clinical events was presented as Kaplan‐Meier estimates. Cox proportional hazard modeling was used to estimate hazard ratio (HR) and 95% confidence interval (CI). The independent association between functional IR (m‐rFSS>0) and 2‐year MACE was examined in a Cox model adjusted for clinical (age, sex, hypertension, diabetes mellitus, hyperlipidemia, family history of coronary artery disease (CAD), previous MI, and presentation with acute coronary syndrome) and angiographic (baseline anatomic SYNTAX score) covariates historically related to MACE. Receiver operating characteristic (ROC) curves analysis with area under the curve (AUC) was used to compare the prediction capability of anatomic rSS, c‐rFSS, and m‐rFSS for 2‐year MACE, and the reclassification ability was assessed using category‐free net reclassification improvement (NRI) and integrated discrimination improvement (IDI). NRI was defined as (p_improved_prediction_event_ + p_improved_prediction_nonevent_) − (p_worsened_prediction_event_ + p_worsened_prediction_nonevent_), indicating the proportion of patients who were correctly reclassified for the predicted probability of 2‐year MACE. IDI was defined as (∑^i^_event_ (p_new_(i) − p_old_(i))/n (event) − (∑^j^_nonevent_ (p_new_(j) − p_old_(j))/n (nonevent)), indicating the average improvement in predicted probability of 2‐year MACE. Compared with the predictive models including traditional clinical risk factors (age, sex, hypertension, diabetes mellitus, hyperlipidemia, family history of CAD, history of myocardial infarction, and acute coronary syndrome), the incremental prognostic values of anatomic rSS, c‐rFSS, and m‐rFSS beyond these clinical risk factors were evaluated by comparison of AUC, the category‐free NRI, and the IDI. Unless otherwise specified, a 2‐sided *p* <0.05 was considered to indicate statistical significance. All statistical analyses were performed using SAS software, version 9.4 (SAS Institute).

## 3 Results

**Figure 2:**
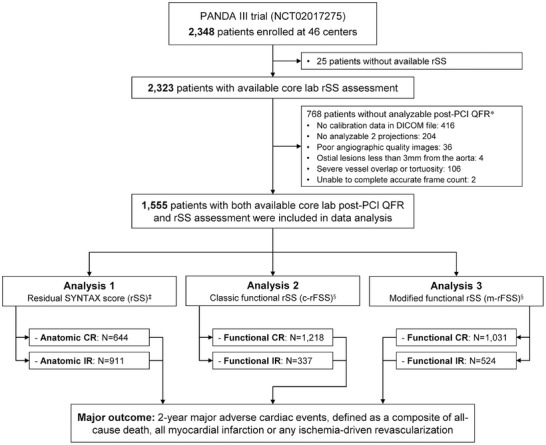
Study Flowchart. *A hierarchical listing based on the processes for QFR assessment was used to identify the exclusion reasons per patient. †Angiographic DICOM files should record the following parameters in order for 3D angiographic reconstruction and QFR computation, 1) distance source to the detector; 2) distance source to patient; and 3) imager pixel spacing and DICOM files were not analyzable if any of the 3 parameters were missing. ‡Complete revascularization was defined as anatomic rSS or classic/modified functional residual SYNTAX score of 0. c‐rFSS = classic residual functional SYNTAX score; DICOM = Digital Imaging and Communication in Medicine; m‐rFSS = modified residual functional SYNTAX score; rSS = residual SYNTAX score; other abbreviations as in Figure 1.

Among the 2348 patients enrolled in the PANDA III trial, 793 patients were excluded because of unavailable core laboratory rFSS calculations, of which 25 were without available rSS and 768 with at least one vessel whose post‐PCI QFR cannot be analyzed (**Figure** **2**). Therefore, 1555 patients were included in the present study (Figure 2). The baseline characteristics of the included and excluded cohorts are shown in Table S2 (Supporting Information). The excluded patients were more likely to have older age, male, diabetes mellitus, and lower creatinine clearance. The two‐year MACE rates were similar between excluded and included patients (log‐rank *p* = 0.26) (Figure S1, Supporting Information). Among the 1555 patients with core laboratory assessments, PCI completeness was determined by rSS, c‐rFSS, and m‐rFSS, respectively, with 644 (41.4%) having anatomic CR (rSS = 0), 1218 (78.3%) reaching functional CR defined as c‐rFSS = 0, and 1031 (66.3%) achieving m‐rFSS‐based functional CR (m‐rFSS = 0) (Figure 2).

### 3.1 Baseline Characteristics

Bassline clinical, angiographic, and procedural characteristics of patients achieving versus not achieving Functional CR (defined as m‐rFSS = 0) are shown in Tables S3 and S4 (Supporting Information). Patients with functional IR (m‐rFSS >0) were more likely to have coexisting comorbidities (e.g., older age, diabetes mellitus, hypertension, chronic kidney disease), and characteristics reflecting the high complexity of the coronary lesions, such as multivessel CAD, left anterior descending (LAD) involvement, total occlusion, bifurcation lesion, severe calcification, diffuse and tandem lesions, and a higher baseline SYNTAX score. Patients with functional IR also had more vessels treated with greater numbers of stents and a higher residual SYNTAX score.

### 3.2 Distributions, Correlation, and Reclassification of Anatomic and Physiological Scoring Systems

Figure S2 (Supporting Information) shows the distributions of rSS, c‐rFSS, m‐rFSS, and post‐PCI QFR as assessed by the core laboratory. The mean rSS, c‐rFSS, and m‐rFSS were 4.3 ± 5.5, 1.2 ± 3.3, 1.7 ± 3.6, respectively. For treated vessels, the mean post‐PCI QFR was 0.95 ± 0.08, respectively. Both c‐rFSS and m‐rFSS were correlated with anatomic rSS, with correlation coefficients (r) of 0.71 and 0.70, respectively (Figure S3, Supporting Information). The c‐rFSS was strongly correlated with m‐rFSS (r = 0.91, *p* < 0.001).

**Figure 3:**
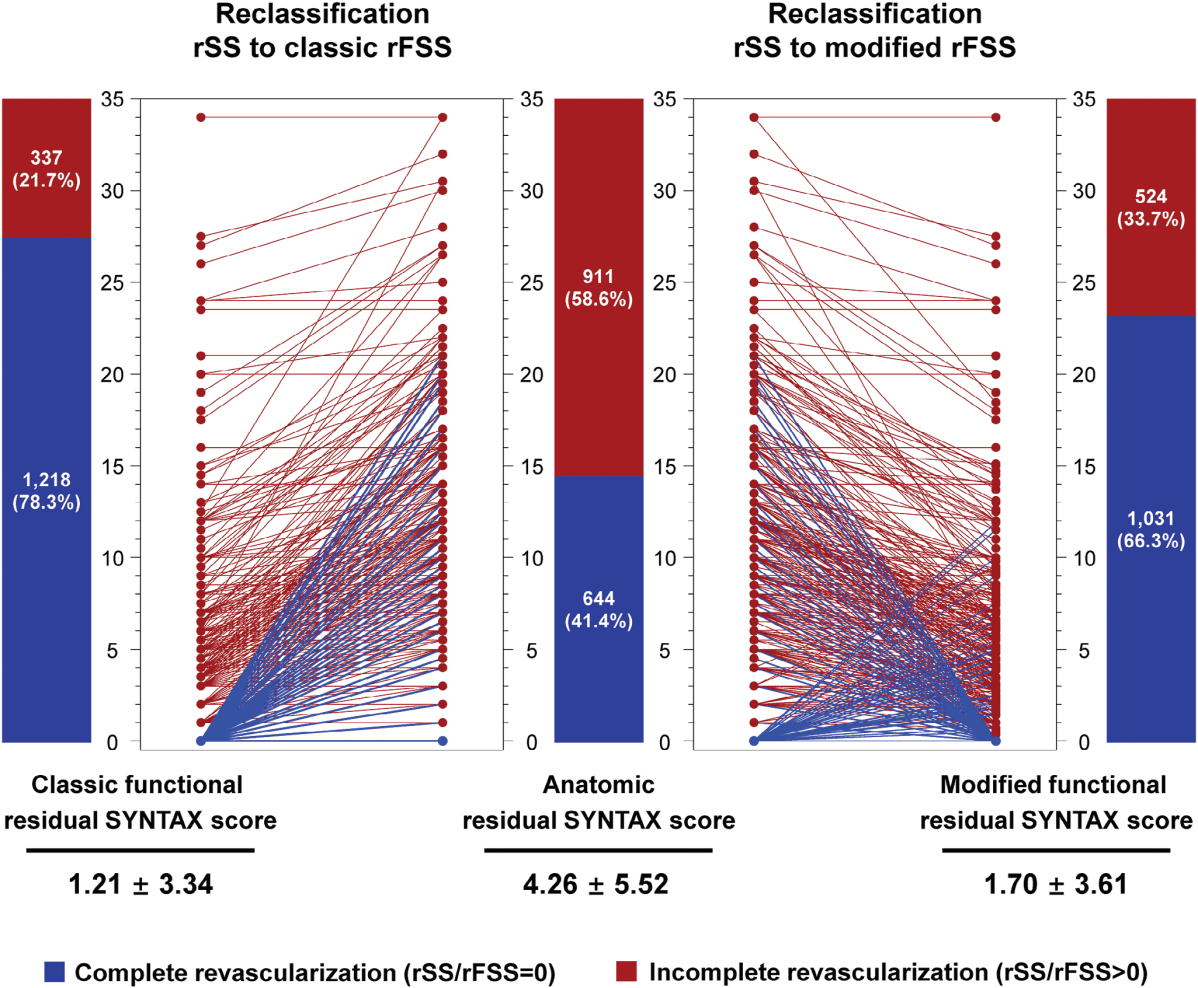
Reclassification by Classic Residual Functional SYNTAX Score and Modified Residual Functional SYNTAX Score. Anatomic CR (rSS = 0) was achieved in 644 (41.4%) patients, while 911 (58.6%) patients had anatomical IR (rSS >0). After the calculation of c‐rFSS, 36.9% of patients were unidirectionally reclassified from anatomic IR to functional CR (c‐rFSS = 0), while bidirectional reclassification was observed after the assessment of m‐rFSS with 31.4% of patients trans‐group from anatomic IR to functional CR (m‐rFSS = 0) and 6.6% of patients being reclassified from anatomic CR to functional IR (m‐rFSS >0). rFSS = residual functional SYNTAX score; other abbreviations as in Figures 1 and 2.

Anatomic CR (rSS = 0) was achieved in 644 (41.4%) patients, while 911 (58.6%) patients had anatomical IR (rSS >0). After the calculation of c‐rFSS, 36.9% (574/1555) of patients were unidirectionally reclassified from anatomic IR to functional CR (c‐rFSS = 0), while bidirectional reclassification was observed after the assessment of m‐rFSS with 31.4% (489/1555) of patients trans‐group from anatomic IR to functional CR (m‐rFSS = 0) and 6.6% (102/1555) of patients being reclassified from anatomic CR to functional IR (m‐rFSS >0) (**Figure** **3**). Among those with c‐rFSS‐based functional CR, 187 (12.0%) had m‐rFSS‐based functional IR (Figure S4, Supporting Information).

### 3.3 Two‐Year Clinical Outcomes

**Figure 4:**
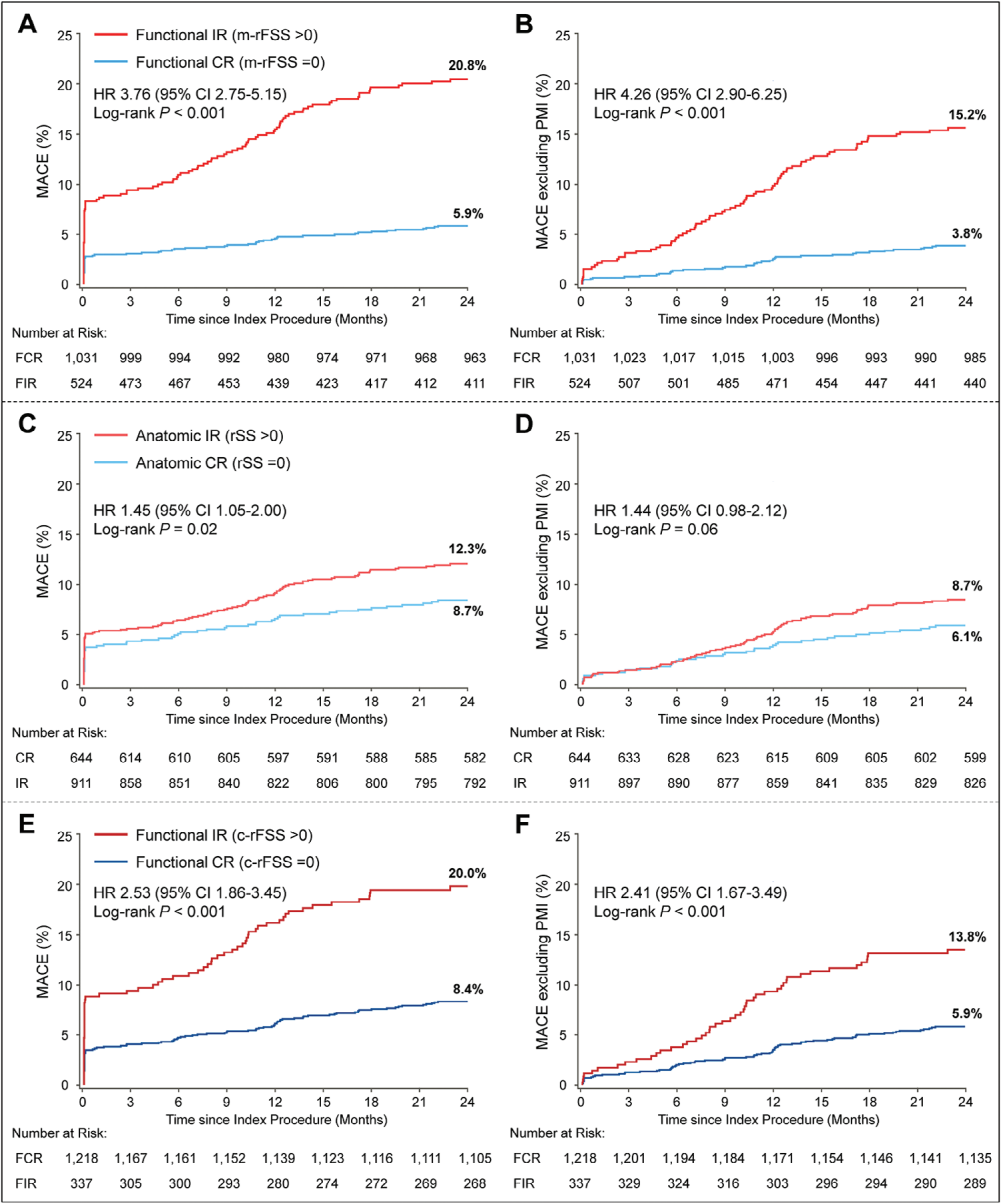
Time‐to‐event curves for 2‐year clinical outcomes stratified by Anatomic or Functional Revascularization Completeness. Kaplan‐Meier time‐to‐first event curves showing the 2‐year cumulative incidences of MACE and MACE excluding PMI by revascularization completeness based on modified residual functional SYNTAX score (A and B), anatomic residual SYNTAX score (C and D) and classic residual functional SYNTAX score (E and F), respectively. CI = confidence interval; HR = hazard ratio; MACE = major adverse composite endpoint; PMI = periprocedural myocardial infarction; other abbreviations as in Figures 1 and 2.

Complete 2‐year follow‐up data were available in 1542 (99.2%) of 1555 patients, with a median follow‐up duration of 728 days (IQR: 715–738). The Kaplan‐Meier estimates for major 2‐year MACE outcomes were 5.9 and 20.8% of patients with m‐rFSS‐based functional CR (m‐rFSS = 0) and functional IR (m‐rFSS >0), respectively (HR 3.76, 95% CI: 2.75–5.15, *p*<0.001) (**Figure** **4**A and **Table** **1**). The 2‐year rate of the major secondary outcome of MACE excluding periprocedural MI was significantly higher in the m‐rFSS‐based functional IR group (15.2% vs 3.8%; HR 4.26, 95% CI: 2.90–6.25) (Figure 4B and Table 1). These differences were mainly driven by higher rates of all‐cause death (4.0% vs 1.4%), MI (9.2% vs 3.2%), and ischemia‐driven revascularization (10.9% vs 2.1%). Those with functional IR also had higher 2‐year event rates of other secondary outcomes, including cardiovascular death, periprocedural MI, any revascularization, and stent thrombosis. The prognosis difference for the 2‐year MACE between the 2 groups was consistent across various subgroups (Figure S5, Supporting Information). In addition, two‐year MACE risk was continuously associated with m‐rFSS value (per 1 point increase, HR 1.07, 95% CI: 1.04–1.09, *p*<0.001) (Figure S6C, Supporting Information). After multivariable adjustment for baseline clinical and angiographic differences, m‐rFSS‐based functional IR was an independent predictor of freedom from 2‐year MACE (adjusted HR 3.32, 95% CI: 2.34–4.71; *p*<0.001) (full model shown in **Table** **2**).

**Table 1:** Two‐year clinical outcomes in patients with functional CR (m‐rFSS = 0) and functional IR (m‐rFSS>0).

	Total *N* = 1555	Functional CR (m‐rFSS = 0) *N* = 1031	Functional IR (m‐rFSS>0) *N* = 524	Hazard Ratio (95% CI)	*p* value
MACE	169 (10.9)	61 (5.9)	108 (20.6)	3.76 (2.75–5.15)	<0.001
All‐cause death	35 (2.3)	14 (1.4)	21 (4.0)	3.00 (1.53–5.91)	0.001
All myocardial infarction	81(5.2)	33 (3.2)	48 (9.2)	2.94 (1.89–4.57)	<0.001
Ischemia‐driven revascularization	79 (5.1)	22 (2.1)	57 (10.9)	5.45 (3.33–8.91)	<0.001
MACE excluding periprocedural MI	118 (7.6)	39 (3.8)	79 (15.1)	4.26 (2.90–6.25)	<0.001
All‐cause death or myocardial infarction	104 (6.7)	43 (4.2)	61 (11.6)	2.89 (1.96–4.28)	<0.001
Other clinical outcomes					
Cardiac death	15 (1.0)	4 (0.4)	11 (2.1)	5.48 (1.74–17.2)	0.004
Periprocedural MI	66 (4.2)	27 (2.6)	39 (7.4)	2.89 (1.77–4.72)	<0.001
Non‐procedural MI	15 (1.0)	6 (0.6)	9 (1.7)	3.01 (1.07–8.46)	0.04
Target vessel related	14 (0.9)	5 (0.5)	9 (1.7)	3.61 (1.21–10.8)	0.02
Any revascularization	82 (5.3)	24 (2.3)	58 (11.1)	5.08 (3.16–8.18)	<0.001
Target vessel revascularization	37 (2.4)	10 (1.0)	27 (5.2)	5.52 (2.67–11.4)	<0.001
Ischemia‐driven	36 (2.3)	9 (0.9)	27 (5.2)	6.14 (2.89–13.1)	<0.001
Non‐target vessel revascularization	50 (3.2)	15 (1.5)	35 (6.7)	4.83 (2.64–8.84)	<0.001
Ischemia‐driven	48 (3.1)	14 (1.4)	34 (6.5)	5.02 (2.70–9.36)	<0.001
Stent thrombosis, definite or probable	15 (1.0)	5 (0.5)	10 (1.9)	3.98 (1.36–11.6)	0.01

Values are Kaplan‐Meier estimated rates, summarized as counts (%). Patients with functional IR were associated with 2‐year poor prognosis.

CI = confidence interval; MACE = major adverse cardiac events; CR = complete revascularization; *N* = number of patients; MI = myocardial infarction; m‐rFSS = modified residual functional SYNATX Score; IR = incomplete revascularization.

**Table 2:** Independent predictors of 2‐year MACE.

	Multivariate variables		
	Adjusted HR	95% CI	*p* Value
Age	1.03	1.01–1.05	<0.001
Sex (male)	1.27	0.89–1.80	0.18
Hypertension	1.17	0.84–1.63	0.36
Diabetes mellitus	1.34	0.96–1.88	0.09
Hyperlipidemia	1.13	0.82–1.56	0.46
Family history of CAD	0.69	0.30–1.58	0.38
Previous MI	1.20	0.82–1.74	0.35
Presentation with ACS	1.02	0.68–1.52	0.93
Baseline anatomic SYNTAX score, per 1 unit	1.00	0.99–1.02	0.77
Functional IR (m‐rFSS>0)	3.32	2.34–4.71	<0.001

After multivariable adjustment for confounders, m‐rFSS‐based functional IR was an independent predictor of 2‐year MACE.

ACS = acute coronary syndrome; CI = confidence interval; HR = hazard ratio; MI = myocardial infarction; other abbreviations as in Table 1.

The results of the Kaplan‐Meier estimates and 2‐year clinical outcomes among patients stratified by anatomic rSS or c‐rFSS were similar to those of m‐rFSS, while the differences between m‐rFSS groups were more pronounced (Figure 4; Tables S5 and S6 and Figure S6, Supporting Information).

### 3.4 Prognostic Values of rSS, c‐rFSS, and m‐rFSS

The ROC curves of rSS, c‐rFSS, and m‐rFSS for predicting 2‐year MACE are shown in Figure S7 (Supporting Information), with AUCs of 0.56 (95% CI: 0.52–0.61), 0.60 (95% CI: 0.55–0.65), and 0.67 (95% CI: 0.62–0.71), respectively. Compared with c‐rFSS, m‐rFSS showed improved reclassification ability (difference in AUC: 0.07, *p* < 0.001; NRI: 0.54, *p* < 0.001; IDI: 0.57%, *p* < 0.001). Sensitivity analysis was performed comparing the predictive ability of m‐rFSS with different weighting factors for QFR value range from 0.80 to 0.90 (i.e., 0.50, 0.60, 0.70, and 0.80), and similar AUC values were observed (Table S7, Supporting Information).

**Figure 5:**
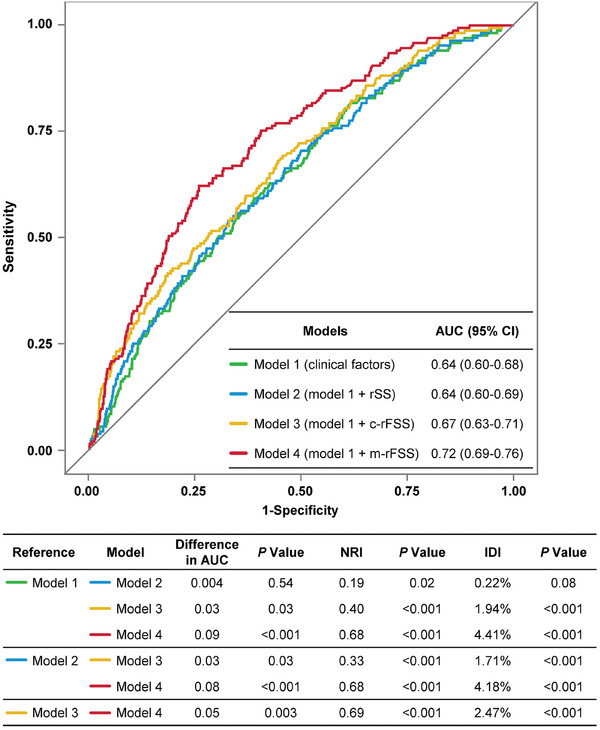
Comparison of Discrimination and Reclassification Ability of Models with rSS, c‐rFSS or m‐rFSS in Addition to Clinical Risk Factors. AUC, NRI, and IDI values of models with rSS, c‐rFSS, or m‐rFSS in addition to clinical risk factors were compared. *The reference model (model 1) included clinical risk factors only, including age, sex, hypertension, diabetes mellitus, hyperlipidemia, family history of CAD, history of myocardial infarction, and acute coronary syndrome. Model 2 included clinical risk factors plus rSS. Model 3 included clinical risk factors plus c‐rFSS. Model 4 included clinical risk factors plus m‐rFSS. AUC = area under the curve; NRI = net reclassification improvement; IDI = integrated discrimination index; other abbreviations as in Figures 1 and 2.

When each of the different scoring systems (i.e., anatomic rSS, c‐rFSS, and m‐rFSS) was added to clinical risk factors for predicting 2‐year MACE, the model with m‐rFSS showed the highest discrimination and reclassification ability (difference in AUC: 0.09, *p* < 0.001; NRI: 0.68, *p* < 0.001; IDI: 4.41%, *p* < 0.001) (**Figure** **5**). Compared with the model with c‐rFSS, the model with m‐rFSS showed statistically improved AUC (difference in AUC: 0.05, *p* = 0.003), NRI (0.69, *p* < 0.001) and IDI (2.47%, *p* < 0.001). All models had acceptable calibration results, as assessed by the Hosmer‐Lemeshow test with *p* values of 0.988, 0.996, 0.511, and 0.308, respectively (Figure S8, Supporting Information).

## 4 Discussion

The present study optimized the algorithms of c‐rFSS regarding its inherent limitations and developed a novel m‐rFSS. The major findings are as follows: 1) achieving functional CR as defined by a m‐rFSS = 0 was strongly associated with freedom from 2‐year MACE, with evident benefits in both the periprocedural period and during late follow‐up; 2) revascularization completeness as determined by m‐rFSS was an independent predictor of MACE in multivariate adjusted model; 3) compared with revascularization completeness classified by anatomic rSS, bidirectional trans‐grouping was observed, i.e., nearly 6.6% of patients with anatomic CR were reclassified as having functional IR and over 30% with anatomic IR were transferred to functional IR group after calculation of m‐rFSS; 4) over one‐fifth of patients with c‐rFSS‐based functional CR were reclassified as having functional IR after m‐rFSS calculation; 5) m‐rFSS was more predictive of 2‐year MACE than that of anatomic rSS or c‐rFSS, with improved reclassification ability; and 6) prognostic models including the m‐rFSS had highest discrimination and reclassification ability than those containing only clinical variables or that combined anatomic rSS, or c‐rFSS.

The anatomic rSS is a comprehensive scoring system that can quantify the residual anatomical burden of lesions after PCI as well as the extent of revascularization completeness.^[^ ^11^ ^]^ Patients who achieve anatomic CR (anatomic rSS = 0) after PCI have improved prognosis compared with those with anatomic IR.^[^ ^10^ ^]^ However, randomized trials of coronary physiology‐guided PCI have demonstrated that deferring treatment of anatomically significant but hemodynamically nonflow‐limiting lesions is associated with a favorable prognosis.^[^ ^27^, ^28^ ^]^ A similar prognosis was observed among patients who achieved functional CR (defer non‐flow‐limiting lesions) regardless of the extent of residual untreated anatomic disease (assessed by anatomic rSS).^[^ ^29^ ^]^ The definition of CR after PCI has moved from the concept of “anatomy” to “physiology” in recent decades.

The c‐rFSS integrates the residual anatomic and functional disease burden and sums only the scores for hemodynamically flow‐limiting lesions.^[^ ^12^ ^]^ Numerous studies have demonstrated that the c‐rFSS can better discriminate the risk of adverse events after PCI and that it can identify patients with functional CR.^[^ ^12^, ^13^ ^]^ In a pre‐specified sub‐analysis of the 3 V FFR‐FRIENDS registry, patients with functional IR (FFR‐based c‐rFSS≥1) had a 4‐fold higher adjusted 2‐year risk of MACE than those with functional CR (FFR‐based c‐rFSS = 0). Recent studies have investigated the feasibility of calculating c‐rFSS based on the computational coronary physiology index.^[^ ^12^ ^]^ In a pre‐specified substudy of the FAVOR III China trial,^[^ ^13^ ^]^ The c‐rFSS was determined by a novel, simple‐to‐use, and validated angiography‐based QFR system. It demonstrated that the achievement of functional CR (defined as QFR‐based c‐rFSS = 0) was associated with a marked (≈75%) reduction in the 1‐year MACE rate. In the present study, our data similarly revealed improved prognosis in patients who achieved functional CR with significant reductions in all individual components of MACE.

The c‐rFSS is calculated only for lesions that are anatomically and physiologically significant (visual DS ≥50%, and post‐PCI FFR or QFR ≤0.80). It can only downgrade severity and may underestimate the prognostic risk in specific populations owing to its underlying scoring rationale. In particular, c‐rFSS ignores the impact of lesions with suboptimal functional results (e.g., post‐PCI FFR or QFR between 0.80 and 0.90) as well as angiographically negative but functionally significant lesions (visual DS <50% but post‐PCI FFR or QFR ≤0.80).

In the present study, we optimized the algorithms of c‐rFSS regarding the aforementioned scenarios and obtained the m‐rFSS as follows: 1) for lesions with suboptimal functional results, an extra physiology weighting of 0.7 was assigned (this was previously 0); 2) for anatomically negative but functionally significant lesions, a multiplication factor of 1 was used (this was previously 0). The functional IR defined by m‐rFSS >0 was associated with a marked (more than 3×) increase in the 2‐year MACE and proved to be an independent risk factor after correcting for differences in baseline clinical characteristics and the extent and complexity of coronary artery disease. Nearly 12% of patients with c‐rFSS‐based functional CR were re‐stratified as having m‐rFSS‐based functional IR, and thus had worse prognosis. Interestingly, compared to anatomic rSS‐based grouping, bidirectional reclassification was observed after the assessment of m‐rFSS with more than 30% of patients with anatomic IR being reclassified as having achieved functional CR and nearly 6.6% of patients with anatomical CR trans‐group to functional IR. In contrast, only unidirectional reclassification was observed when c‐rFSS was used, indicating that the c‐rFSS can identify only low‐risk patients whereas the m‐rFSS can discriminate not only low‐risk but also high‐risk patients. Thus, the m‐rFSS (AUC 0.67) was more predictive of 2‐year MACE than that of anatomic rSS (AUC 0.56) and c‐rFSS (AUC 0.60), and it showed improved reclassification ability (significant NRI and IDI). Moreover, adding information from the m‐rFSS led to supreme predictive accuracy and discrimination ability compared with models that contained only clinical variables and with models that add information from the anatomic rSS or c‐rFSS.

As case examples are shown in Figure 1, post‐PCI incomplete revascularization was identified in this case by calculating the m‐rFSS, attributed to residual disease of LAD and LCX. Based on anatomical rSS, incomplete revascularization was also identified but ascribed to LCX and RCA. In contrast, this case was recognized as having complete revascularization by c‐rFSS. Thus, when introducing m‐rFSS in real‐world clinical scenarios, more precise post‐PCI risk stratification and identification of “responsible vessels” would achieve, thereby guiding further optimization of treatment and management strategies (e.g., extra interventions, intensive medication, and care).

In recent decades, increasing emphasis has been placed on the prognostic impact of post‐PCI suboptimal functional results, suggesting that suboptimal functional results (e.g., post‐PCI FFR or QFR ≤0.90) are prognostically adverse despite the absence of hemodynamically flow‐limiting (post‐PCI FFR or QFR >0.80).^[^ ^18^, ^19^, ^20^, ^21^ ^]^ In particular, the latest studies have confirmed that suboptimal functional results determined by computational physiological index (QFR) were associated with worse outcomes.^[^ ^20^, ^21^, ^30^ ^]^ In this study, nearly 19.7% (306/1555) of patients had lesions with post‐PCI QFR range from 0.80 to 0.90; thus, 12.0% (187/1555) were further stratified from c‐rFSS‐based functional CR to m‐rFSS‐based functional IR. Mismatch between coronary anatomic and physiological assessments are not infrequent.^[^ ^14^, ^15^, ^16^, ^17^, ^18^ ^]^ Previous studies showed more than 14% of angiographically mild (DS <50%) lesions were hemodynamically significant (FFR ≤0.80), and demonstrated that the angiographic appearance is less important than hemodynamic significance in terms of prognosis.^[^ ^14^ ^]^ A latest post‐PCI QFR study revealed that nearly 11.1% of patients presented with such residual disease after LM bifurcation stenting, and was associated with poor prognosis.^[^ ^17^ ^]^

In the present study, ≈8.7% (136/1555) of patients had lesions with post‐PCI visual DS <50% but QFR ≤0.90 (4.0% for visual DS <50% but QFR ≤0.80); 6.9% (108/1555) of these patients were further reclassified from c‐rFSS‐based functional CR to m‐rFSS‐based functional IR.

In the last few years, an increasing number of factors have been shown to correlate with long‐term prognosis after PCI and are potentially significant prognostic factors, including clinical risk factors (e.g., age, sex, diabetes mellitus, geographic disparity, LVEF, and chronic obstructive pulmonary disease), anatomical risk factors (e.g., SYNTAX score, lesion location, calcification, bifurcation lesion), preprocedural biological markers (e.g., creatinine clearance, C‐reactive protein, HbA1c, hemoglobin, genetic predispositions), procedural and post‐procedural risk factors (e.g., stent length and diameters, optimal medical therapy, completeness of revascularization).^[^ ^31^ ^]^ Numerous scores and prediction models combined coronary disease burden with these prognostic factors for a more comprehensive and individualized assessment, which allowed for an accurate individualized prediction of prognosis. The SYNTAX score II^[^ ^32^ ^]^ and redeveloped SYNTAX score II 2020^[^ ^33^ ^]^ combined anatomic SYNTAX score and prognostic factors showed better discrimination and calibration for outcome risk. This study remains anchored in the traditional scoring algorithm, focusing exclusively on coronary anatomy and physiology. Although a prognostic model combining the m‐rFSS and clinical risk factors was also developed in this study and showed good discrimination and reclassification ability, the model incorporated only a limited number of risk factors (as the original PANDA III study did not collect sufficiently many metrics) and lacked of comprehensive and individualized. Future prospective research with pre‐set comprehensive data collection plan would further incorporate coronary anatomy, coronary physiology, and a wide range of risk factors to establish prognostic models using machine learning techniques.^[^ ^34^ ^]^ In addition, the development of artificial intelligence (AI)‐powered novel coronary analysis and Computed Tomography (CT)‐derived FFR in recent years is expected to promote more simple, accurate, faster, and non‐invasive coronary physiology assessments.^[^ ^17^, ^35^, ^36^, ^37^ ^]^

## 5 Limitations

First, this is a proof‐of‐concept study that retrospectively analyzed a published randomized research cohort. Thus, the treatment and optimization strategy was left to the interventionalists’ discretion and had been previously completed. The impact of further optimization strategies for patients with functional IR could not be evaluated. Prospective research is needed to determine the extent to which routine online post‐PCI physiological assessment with subsequent optimization may improve long‐term prognosis. Second, due to the accessibility of resources and the fact that the calculation of m‐rFSS is predicated on the necessity of a QFR or other physiological assessment, there was no appropriate dataset for external validation, which would leave uncertainty about the generalizability of the present result. Third, the quality of angiographic images would affect the acquirement and accuracy of both anatomical features (e.g., diameter stenosis, lesion location) and computational physiology index (QFR), which would impact the results of m‐rFSS. The present study was a *post‐hoc* analysis of the PANDA III trial in which angiograms were not acquired with pre‐set specific guidelines. Thus, many angiographic images failed to meet the analysis requirements, resulting in nearly one‐third of patients being excluded, in line with previous studies.^[^ ^21^ ^]^ Some baseline characteristics were not evenly distributed between included and excluded patients. In addition, there was a risk of overfitting considering retrospective design and single‐dataset analysis. The extent to which these considerations may affect the external validity of the present results is unknown. Fourth, a recent anonymous comparison by an independent core lab showed only modest diagnostic accuracy for angiography‐derived FFR methodologies compared to invasive FFR.^[^ ^38^ ^]^ These may affect the accuracy of the QFR‐derived rFSS since rFSS is originally based on invasive FFR. Fifth, some baseline characteristics of patients between m‐rFSS‐based functional CR and IR groups were imbalanced. Although the prognostic implication of having functional IR persisted after multivariable adjustment for these differences, the impact of unmeasured confounders cannot be discounted. Moreover, associations do not prove causality, so our findings should be considered hypothesis‐generating. Sixth, although a prognostic model combining m‐rFSS and clinical risk factors was developed in this study and showed good predictive ability of 2‐year prognosis, many of the potentially significant prognostic factors could not be incorporated due to the retrospective analysis. Future prospective researches were needed to combine coronary anatomy, coronary physiology, and a wide range of risk factors to establish prognostic models based on advanced methods (e.g., machine learning). Seventh, rFSS calculation was performed offline in a core laboratory. Site‐assessed and core laboratory–determined SS values may vary. Seventh, the use of intracoronary imaging was relatively infrequent in the PANDA III trial in which the underlying cause of functional IR could not be investigated, and the extent to which the present results may have differed due to a greater reliance on intracoronary imaging is uncertain. Finally, although higher prognostic significance of the m‐rFSS was observed in this *post hoc* analysis of the PANDA III trial with acceptable sample sizes, the actual clinical benefits of this scoring system should be further validated in a larger population due to modified factors (e.g., *modified* *W_P_*, *S_modified_*) obtained based on data from original PANDA III trial.

## 6 Conclusion

In this study, we optimized the classic scoring algorithm to develop a novel scoring system (m‐rFSS), and revascularization completeness determined by m‐rFSS was markedly associated with a 2‐year prognosis. Prognostic models including the m‐rFSS were substantially more accurate than those containing only clinical variables or those combined anatomic rSS or c‐rFSS.

## Conflict of Interest

The authors declare no conflict of interest.

## Author Contributions

R.Z., S.W., and Q.L. contributed equally to this work. All authors have participated in the work. K.D. had full access to all of the data in the study and took responsibility for the integrity of the data and the accuracy of the data analysis. K.D. and D.Y. contributed to the conception or design of the work. R.Z., S.W., Q.L., C.G., H.W., S.Y., L.X., Y.H., Z.Q., R.F., L.F., C.Z., and L.S. contributed to the acquisition of data for the work. R.Z., S.W., and W.L. contributed to the statistical analysis of the data. R.Z., S.W., and Q.L. drafted the manuscript. K.D., D.Y., and L.S. critically revised the manuscript. K.D. and D.Y. finalized the manuscript. All authors reviewed the manuscript.

## Supporting information



Supporting Information

## Data Availability

The data that support the findings of this study are available on request from the corresponding author. The data are not publicly available due to privacy or ethical restrictions.
