# Supplementary material for: Functional Complete Revascularization as Determined by an Optimized Scoring System After Revascularization: A Post Hoc Analysis from Multi‐Center PANDA III Trial
Source: Adv Sci (Weinh). 2025 Feb 18;12(14):2415961. doi: 10.1002/advs.202415961 (PMC11984873; doi:10.1002/advs.202415961)

**SUPPLEMENTARY APPENDIX**

**Functional complete revascularization as determined by an optimized scoring system after revascularization: A post hoc analysis from multi-center PANDA III trial**

| **Table of Contents** | | **Page Number** |
| --- | --- | --- |
| Methods | Detailed Methodology of Determination of ${modified W}_{P}$ | 2 |
| Supplemental Table 1 | Key Differences among rSS, c-rFSS, and m-rFSS | 3 |
| Supplemental Table 2 | Baseline Characteristics of m-rFSS Analyzable and Unanalyzable Patients | 4 |
| Supplemental Table 3 | Baseline Characteristics of Study Population by Modified Residual Functional SYNTAX Score | 5 |
| Supplemental Table 4 | Angiographic and Procedural Characteristics of Study Population by Modified Residual Functional SYNTAX Score | 6 |
| Supplemental Table 5 | Two-year Clinical Outcomes in Patients with Functional CR (c-rFSS=0) and Functional IR (c-rFSS>0) | 8 |
| Supplemental Table 6 | Two-year Clinical Outcomes in Patients with Anatomic CR (rSS=0) and Anatomic IR (rSS>0) | 9 |
| Supplemental Table 7 | Predictive Ability of 2-year MACE for m-rFSS with Different Modified Physiology Weighting (i.e., 0.50, 0.60, 0.70 and 0.80) | 10 |
| Supplemental Figure 1 | Time-to-Event Curves of 2-Year MACE in Analyzable and Unanalyzable Groups | 11 |
| Supplemental Figure 2 | Distribution of rSS, c-rFSS, m-rFSS and Post-PCI QFR | 12 |
| Supplemental Figure 3 | Correlations Among rSS, c-rFSS, and m-rFSS | 13 |
| Supplemental Figure 4 | Reclassification by Modified Residual Functional SYNTAX Score | 14 |
| Supplemental Figure 5 | Subgroup analyses | 15 |
| Supplemental Figure 6 | Association Between the Estimated risk of Clinical Events and continuous rSS, c-rFSS or m-rFSS | 16 |
| Supplemental Figure 7 | Receiver-operating characteristic curve of Anatomic rSS, c-rFSS, or m-rFSS for predicting 2-year MACE | 17 |
| Supplemental Figure 8 | Calibration Plots of Models with rSS, c-rFSS or m-rFSS in Addition to Clinical Risk Factors | 18 |

**METHODS**

**Detailed Methodology of Determination of** ${\boldsymbol{modified}\boldsymbol{W}}_{\boldsymbol{P}}$

Multivariate Cox regression analysis was used to estimate the adjusted hazard ratios (HR) among different post-PCI physiological results (i.e., post-PCI QFR ≤0.80, 0.80-0.90, and >0.90), and determine the specific values of ${modified W}_{P}$. A total of 1,930 treated vessels (1,555 patients) with analyzable post-PCI QFR were included in this analysis. In this per-vessel analysis, the outcome was the vessel-oriented composite endpoint (VOCE) at 2 years, defined as a composite of vessel-related cardiac death, vessel-related non-procedural myocardial infarction (MI), and ischemia-driven target vessel revascularization (TVR). The candidate variables were selected according to previous publications, which identified independent predictors including post-PCI FFR or post-PCI QFR of adverse events. Finally, a total of 14 variables were entered into multivariate analysis, i.e., age, male, hypertension, hyperlipidemia, diabetes mellitus, family history of coronary artery disease, peripheral artery disease, acute coronary syndrome, LAD stenosis, tandem lesion, baseline diameter stenosis, baseline lesion length per vessel, total stent length per vessel, and post-PCI physiological results (i.e., QFR ≤0.80, 0.80-0.90, and >0.90). In multivariate analysis, after adjusting for potential confounders, the adjusted HR for vessels with suboptimal physiological results (QFR between 0.80 to 0.90) was 0.70 compared with vessels presented with residual ischemia (QFR ≤0.80), indicating the risk of 2-year VOCE for vessels with suboptimal results reduced by 30% compared with ischemic vessels. Therefore, the ${modified W}_{P}$ was determined as 0.70 for lesions with suboptimal post-PCI results (QFR 0.80-0.90).

**Supplemental Table 1. Key Differences among rSS, c-rFSS, and m-rFSS**

|  | **The rSS** | **The c-rFSS** | **The m-rFSS** |
| --- | --- | --- | --- |
| Coronary Anatomy | ✔ | ✔ | ✔ |
| Coronary Physiology | ✖ | ✔ | ✔ |
| Ischemia (QFR ≤0.80) | ✖ | ✔ | ✔ |
| Suboptimal (QFR 0.80-0.90) | ✖ | ✖ | ✔ |
| Anatomy-physiology Mismatch | ✖ | ✖ | ✔ |
| Clinical Risk Factors | ✖ | ✖ | ✖ |

QFR = quantitative flow ratio; rSS = residual SYNTAX score; c-rFSS = classic residual functional SYNTAX score; m-rFSS = modified residual functional SYNTAX score.

**Supplemental Table 2. Baseline Characteristics of m-rFSS Analyzable and Unanalyzable Patients**

|  | **Total**  **N=2,348** | **m-rFSS Analyzable**  **N=1,555** | **m-rFSS Unanalyzable**  **N=793** | ***P* Value** |
| --- | --- | --- | --- | --- |
| Age, yrs | 61.2 ± 10.7 | 60.7 ± 10.7 | 62.0 ± 10.5 | 0.005 |
| Male | 1,658 (70.6) | 1,076 (69.2) | 582 (73.4) | 0.04 |
| Body mass index, kg/m^2^ | 24.9 ± 3.4 | 24.9 ± 3.4 | 24.8 ± 3.3 | 0.78 |
| Diabetes mellitus | 570 (24.3) | 349 (22.4) | 221 (27.9) | 0.004 |
| Hypertension | 1,447 (61.6) | 950 (61.1) | 497 (62.7) | 0.47 |
| Hyperlipidemia | 732 (31.2) | 497 (32.0) | 235 (29.6) | 0.26 |
| Smoking history | 1,179 (50.2) | 789 (50.7) | 390 (49.2) | 0.49 |
| Family history of coronary artery disease | 117 (5.0) | 81 (5.2) | 36 (4.5) | 0.55 |
| Previous myocardial infarction | 437 (18.6) | 276 (17.7) | 161 (20.3) | 0.15 |
| Previous PCI | 282 (12.0) | 173 (11.1) | 109 (13.7) | 0.07 |
| Previous stroke | 268 (11.4) | 167 (10.7) | 101 (12.7) | 0.15 |
| Peripheral artery disease | 71 (3.0) | 47 (3.0) | 24 (3.0) | 1.00 |
| Creatinine clearance, ml/min | 90.7 ± 44.9 | 92.5 ± 50.1 | 87.1 ± 32.1 | 0.006 |
| Left ventricular ejection fraction, % | 59.3 ± 9.0 | 59.4 ± 8.8 | 59.3 ± 9.4 | 0.78 |
| Acute coronary syndrome | 1,923 (81.9) | 1,275 (82.0) | 648 (81.7) | 0.87 |

Values are mean ± SD or n (%).

CR = complete revascularization; N = number of patients; m-rFSS = modified residual functional SYNATX Score; PCI = percutaneous coronary intervention.

**Supplemental Table 3. Baseline Characteristics of Study Population by Modified Residual Functional SYNTAX Score**

|  | **Total**  **N=1,555** | **Functional CR**  **(m-rFSS=0)**  **N=1,031** | **Functional IR**  **(m-rFSS>0)**  **N=524** | ***P* Value** |
| --- | --- | --- | --- | --- |
| Age, yrs | 60.7 ± 10.7 | 60.1 ± 10.6 | 61.9 ± 10.6 | 0.001 |
| Male | 1,076 (69.2) | 701 (68.0) | 375 (71.6) | 0.16 |
| Body mass index, kg/m^2^ | 24.9 ± 3.4 | 24.9 ± 3.4 | 24.8 ± 3.3 | 0.38 |
| Diabetes mellitus | 349 (22.4) | 212 (20.6) | 137 (26.1) | 0.01 |
| Use of insulin | 106 (6.8) | 68 (6.6) | 38 (7.3) | 0.67 |
| Hypertension | 950 (61.1) | 604 (58.6) | 346 (66.0) | 0.005 |
| Hyperlipidemia | 497 (32.0) | 314 (30.5) | 183 (34.9) | 0.08 |
| Smoking history | 789 (50.7) | 518 (50.2) | 271 (51.7) | 0.59 |
| Family history of coronary artery disease | 81 (5.2) | 50 (4.8) | 31 (5.9) | 0.40 |
| Previous myocardial infarction | 276 (17.7) | 168 (16.3) | 108 (20.6) | 0.04 |
| Previous PCI | 173 (11.1) | 107 (10.4) | 66 (12.6) | 0.20 |
| Previous stroke | 167 (10.7) | 104 (10.1) | 63 (12.0) | 0.26 |
| Peripheral artery disease | 47 (3.0) | 28 (2.7) | 19 (3.6) | 0.35 |
| Creatinine clearance, ml/min | 92.5 ± 50.1 | 94.6 ± 57.0 | 88.3 ± 32.1 | 0.02 |
| Left ventricular ejection fraction, % | 59.4 ± 8.8 | 59.6 ± 8.4 | 58.9 ± 9.4 | 0.12 |
| Clinical presentation |  |  |  | 0.04 |
| Asymptomatic ischemia | 59 (3.8) | 36 (3.5) | 23 (4.4) |  |
| Stable angina | 221 (14.2) | 149 (14.5) | 72 (13.7) |  |
| Unstable angina | 815 (52.4) | 564 (54.7) | 251 (47.9) |  |
| NSTEMI | 227(14.6) | 135 (13.1) | 92 (17.6) |  |
| STEMI | 233 (15.0) | 147 (14.3) | 86 (16.4) |  |

Values are mean ± SD or n (%).

CR = complete revascularization; N = number of patients; m-rFSS = modified residual functional SYNATX Score; NSTEMI: non–ST-segment elevation myocardial infarction; IR = incomplete revascularization; PCI = percutaneous coronary intervention; rFSS = residual functional SYNTAX score; STEMI = ST-segment elevation myocardial infarction.

**Supplemental Table 4. Angiographic and Procedural Characteristics of Study Population** **by Modified Residual Functional SYNTAX Score**

|  | **Total**  **N=1,555** | **Functional CR**  **(m-rFSS=0)**  **N=1,031** | **Functional IR**  **(m-rFSS>0)**  **N=524** | ***P* Value** |
| --- | --- | --- | --- | --- |
| **Per patient** | 1,555 | 1,031 | 524 |  |
| Anatomic SYNTAX score | 13.7 ± 8.9 | 10.8 ± 7.0 | 19.4 ± 9.4 | <0.001 |
| Multivessel CAD | 795 (51.1) | 380 (36.9) | 415 (79.2) | <0.001 |
| Transradial approach | 1,477 (95.0) | 988 (95.8) | 489 (93.3) | 0.04 |
| Treated vessel per patient | 1.24 ± 0.48 | 1.21 ± 0.45 | 1.30 ± 0.53 | 0.001 |
| Stents per patients | 1.68 ± 0.90 | 1.60 ± 0.86 | 1.84 ± 0.96 | <0.001 |
| Total Stent length per patient, mm | 41.5 ± 25.5 | 39.0 ± 24.2 | 46.6 ± 27.3 | <0.001 |
| Residual SYNTAX score (rSS) |  |  |  |  |
| Anatomic rSS | 4.26 ± 5.52 | 2.54 ± 3.63 | 7.65 ± 6.88 | <0.001 |
| Classic functional rSS | 1.21 ± 3.34 | 0 ± 0 | 3.58 ± 4.96 | <0.001 |
| Modified functional rSS | 1.70 ± 3.61 | 0 ± 0 | 5.05 ± 4.66 | <0.001 |
| **Per treated vessel** | 1,930 | 1145 | 785 |  |
| Vessel location |  |  |  |  |
| Left anterior descending artery | 955 (49.5) | 606 (52.9) | 349 (44.5) | <0.001 |
| Left circumflex artery/ramus | 413 (21.4) | 244 (21.3) | 169 (21.5) | 0.95 |
| Right coronary artery | 559 (29.0) | 294 (25.7) | 265 (33.8) | <0.001 |
| Total occlusion | 330 (17.1) | 147 (12.8) | 183 (23.3) | <0.001 |
| Bifurcation lesion | 759 (39.3) | 480 (41.9) | 279 (35.5) | 0.006 |
| Severe distortion | 61 (3.2) | 38 (3.3) | 23 (2.9) | 0.73 |
| Severe calcification | 91 (4.7) | 29 (2.5) | 62 (7.9) | <0.001 |
| Diffusion lesion | 922 (47.8) | 476 (41.6) | 446 (56.8) | <0.001 |
| Tandem lesion* | 419 (21.7) | 156 (13.6) | 263 (33.5) | <0.001 |
| Pre-procedural QCA |  |  |  |  |
| Reference vessel diameter, mm | 2.75 ± 0.47 | 2.80 ± 0.46 | 2.68 ± 0.47 | <0.001 |
| Minimum lumen diameter, mm | 0.66 ± 0.47 | 0.73 ± 0.47 | 0.56 ± 0.45 | <0.001 |
| Diameter stenosis, % | 74.7 ± 16.4 | 72.7 ± 15.9 | 77.7 ± 16.6 | <0.001 |
| Total lesion length per vessel, mm | 22.0 ± 13.3 | 20.9 ± 12.6 | 23.6 ± 14.1 | <0.001 |
| Procedural information |  |  |  |  |
| Balloon pre-dilation | 1,776 (92.7) | 1,043 (91.8) | 733 (94.1) | 0.07 |
| Stents per vessel | 1.41 ± 0.65 | 1.35 ± 0.61 | 1.49 ± 0.70 | <0.001 |
| Total stent length per vessel, mm | 34.7 ± 19.5 | 32.6 ± 17.8 | 37.8 ± 21.3 | <0.001 |
| Balloon post-dilation | 1,067 (55.7) | 632 (55.6) | 435 (55.8) | 0.97 |
| Post-procedural QCA |  |  |  |  |
| In-stent RVD, mm | 2.77 ± 0.45 | 2.82 ± 0.45 | 2.70 ± 0.44 | <0.001 |
| In-stent MLD, mm | 2.53 ± 0.42 | 2.58 ± 0.43 | 2.45 ± 0.41 | <0.001 |
| In-stent diameter stenosis, % | 8.4 ± 5.5 | 8.1 ± 5.3 | 8.8 ± 5.8 | 0.003 |
| Physiological Index |  |  |  |  |
| Pre-PCI QFR | 0.62 ± 0.18 (1,874) | 0.66 ± 0.18  (1,104) | 0.56 ± 0.18  (770) | <0.001 |
| Post-PCI QFR | 0.95 ± 0.08 | 0.98 ± 0.02 | 0.92 ± 0.11 | <0.001 |

Values are mean ± SD or n (%). ^*^More or 2 lesions per vessel.

CAD = coronary artery disease; MLA = minimum lumen diameter; QCA = quantitative coronary angiography; QFR = quantitative flow ratio; rSS = residual SYNTAX score; RVD = reference vessel diameter; other abbreviations as in Table S3.

**Supplemental Table 5. Two-year Clinical Outcomes in Patients with Functional CR (c-rFSS=0) and Functional IR (c-rFSS>0)**

|  | **Total**  **N=1,555** | **Functional CR**  **(c-rFSS=0)**  **N=1218** | **Functional IR**  **(c-rFSS>0)**  **N=337** | **Hazard Ratio**  **(95% CI)** | ***P* Value** |
| --- | --- | --- | --- | --- | --- |
| MACE | 169 (10.9) | 102 (8.4) | 67 (19.9) | 2.44 (1.79-3.32) | <0.001 |
| All-cause death | 35 (2.3) | 21 (1.7) | 14 (4.2) | 2.18 (1.11-4.31) | 0.02 |
| All myocardial infarction | 81(5.2) | 49 (4.0) | 32 (9.5) | 2.33 (1.491-3.65) | <0.001 |
| Ischemia-driven revascularization | 79 (5.1) | 47 (3.9) | 32 (9.5) | 2.50 (1.59-3.92) | <0.001 |
| MACE excluding periprocedural MI | 118 (7.6) | 72 (5.9) | 46 (13.6) | 2.41 (1.67-3.49) | <0.001 |
| All-cause death or myocardial infarction | 104 (6.7) | 64 (5.3) | 40 (11.9) | 2.21 (1.49-3.29) | <0.001 |
| Other clinical outcomes |  |  |  |  |  |
| Cardiac death | 15 (1.0) | 9 (0.7) | 6 (1.8) | 2.22 (0.79-6.26) | 0.13 |
| Periprocedural MI | 66 (4.2) | 38 (3.1) | 28 (8.3) | 2.60 (1.60-4.25) | <0.001 |
| Non-procedural MI | 15 (1.0) | 11 (0.9) | 4 (1.2) | 1.28 (0.41-4.02) | 0.68 |
| Target vessel related | 14 (0.9) | 10 (0.8) | 4 (1.2) | 1.39 (0.43-4.43) | 0.58 |
| Any revascularization | 82 (5.3) | 49 (4.0) | 33 (9.8) | 2.49 (1.60-3.88) | <0.001 |
| Target vessel revascularization | 37 (2.4) | 29 (2.4) | 8 (2.4) | 0.95 (0.43-2.09) | 0.90 |
| Ischemia-driven | 36 (2.3) | 28 (2.3) | 8 (2.4) | 0.98 (0.45-2.16) | 0.97 |
| Non-target vessel revascularization | 50 (3.2) | 23 (1.9) | 27 (8.0) | 4.37 (2.50-7.63) | <0.001 |
| Ischemia-driven | 48 (3.1) | 22 (1.8) | 26 (7.7) | 4.35 (2.46-7.69) | <0.001 |
| Stent thrombosis, definite or probable | 15 (1.0) | 10 (0.8) | 5 (1.5) | 1.70 (0.58-4.98) | 0.34 |

Values are Kaplan-Meier estimated rates, summarized as counts (%).

CI = confidence interval; CR = complete revascularization; c-rFSS = classic residual functional SYNTAX score; MACE = major adverse cardiac events; MI = myocardial infarction; IR = incomplete revascularization.

**Supplemental Table 6. Two-year Clinical Outcomes in Patients with Anatomic CR (rSS=0) and Anatomic IR (rSS>0)**

|  | **Total**  **N=1,555** | **Anatomical CR**  **(rSS=0)**  **N=644** | **Anatomical IR**  **(rSS>0)**  **N=911** | **Hazard Ratio**  **(95% CI)** | ***P* Value** |
| --- | --- | --- | --- | --- | --- |
| MACE | 169 (10.9) | 56 (8.7) | 113 (12.4) | 1.39 (1.01-1.91) | 0.046 |
| All-cause death | 35 (2.3) | 11 (1.7) | 24 (2.6) | 1.40 (0.68-2.86) | 0.36 |
| All myocardial infarction | 81(5.2) | 29 (4.5) | 52 (5.7) | 1.22 (0.78-1.93) | 0.38 |
| Ischemia-driven revascularization | 79 (5.1) | 27 (4.2) | 52 (5.7) | 1.34 (0.84-2.14) | 0.22 |
| MACE excluding periprocedural MI | 118 (7.6) | 39 (6.1) | 79 (8.7) | 1.44 (0.98-2.12) | 0.06 |
| All-cause death or myocardial infarction | 104 (6.7) | 36 (5.6) | 68 (7.5) | 1.27 (0.84-1.90) | 0.25 |
| Other clinical outcomes |  |  |  |  |  |
| Cardiac death | 15 (1.0) | 8 (1.2) | 7 (0.8) | 0.59 (0.21-1.64) | 0.31 |
| Periprocedural MI | 66 (4.2) | 22 (3.4) | 44 (4.8) | 1.36 (0.81-2.28) | 0.24 |
| Non-procedural MI | 15 (1.0) | 7 (1.1) | 8 (0.9) | 0.78 (0.28-2.15) | 0.63 |
| Target vessel related | 14 (0.9) | 7 (1.1) | 7 (0.8) | 0.67 (0.23-1.92) | 0.46 |
| Any revascularization | 82 (5.3) | 27 (4.2) | 55 (6.0) | 1.43 (0.90-2.26) | 0.13 |
| Target vessel revascularization | 37 (2.4) | 18 (2.8) | 19 (2.1) | 0.70 (0.37-1.35) | 0.29 |
| Ischemia-driven | 36 (2.3) | 18 (2.8) | 18 (2.0) | 0.67 (0.35-1.28) | 0.22 |
| Non-target vessel revascularization | 50 (3.2) | 10 (1.6) | 40 (4.4) | 2.83 (1.41-5.67) | 0.003 |
| Ischemia-driven | 48 (3.1) | 10 (1.6) | 38 (4.2) | 2.66 (1.33-5.36) | 0.006 |
| Stent thrombosis, definite or probable | 15 (1.0) | 8 (1.2) | 7 (0.8) | 0.58 (0.21-1.60) | 0.29 |

Values are Kaplan-Meier estimated rates, summarized as counts (%).

CI = confidence interval; CR = complete revascularization; rSS = residual SYNTAX score; MACE = major adverse cardiac events; MI = myocardial infarction; IR = incomplete revascularization.

**Supplemental Table 7. Predictive Ability of 2-year MACE for m-rFSS with Different Modified Physiology Weighting (i.e., 0.50, 0.60, 0.70 and 0.80)**

|  | **AUC** | **95% CI** | ***P* Value** |
| --- | --- | --- | --- |
| The m-rFSS with W_P_ set to 0.50 | 0.668 | 0.624-0.712 | <0.001 |
| The m-rFSS with W_P_ set to 0.60 | 0.668 | 0.624-0.712 | <0.001 |
| The m-rFSS with W_P_ set to 0.70 | 0.669 | 0.624-0.713 | <0.001 |
| The m-rFSS with W_P_ set to 0.80 | 0.668 | 0.624-0.712 | <0.001 |

AUC = area under the curve; CI = confidence interval; MACE = major adverse composite endpoint; m-rFSS = modified residual functional SYNATX Score; W_P_ = modified physiology weighting.

**Supplemental Figure 1. Time-to-Event Curves of 2-Year MACE in Analyzable and Unanalyzable Groups**

MACE = major adverse cadiac endpoint.


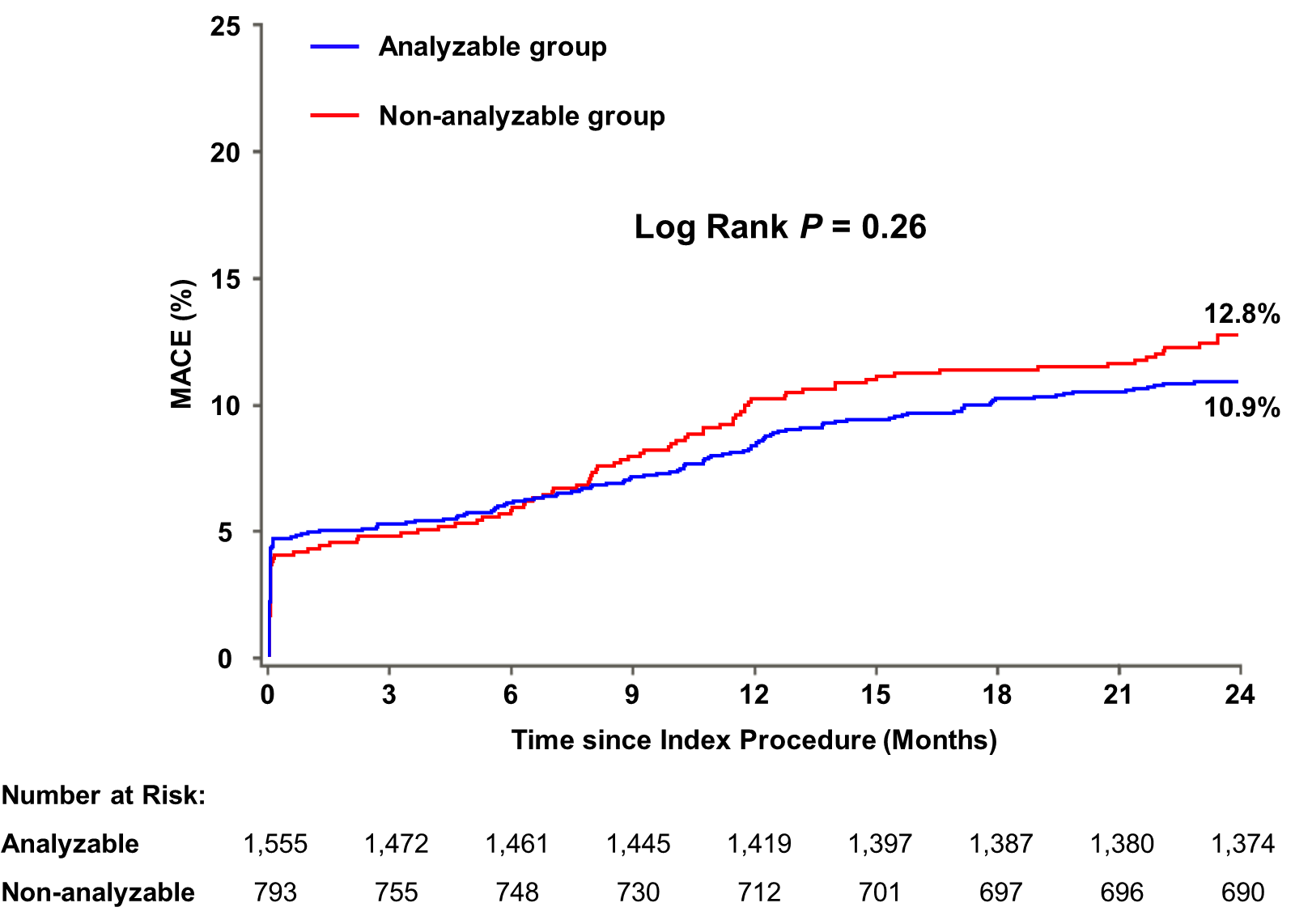


**Supplemental Figure 2. Distribution of rSS, c-rFSS, m-rFSS and Post-PCI QFR**

Distribution of anatomic residual SYNTAX score (A), classic residual functional SYNTAX score (B), modified residual functional SYNTAX score (C), and post-PCI QFR (D).

Np = number of patients; Nv = number of vessels; rFSS = residual functional SYNTAX

score; rSS = residual SYNTAX score; PCI = percutaneous coronary intervention; Q1 = first quartile; Q3 = third quartile; QFR = quantitative flow ratio; other abbreviations as in Figures S1 and S2.

**
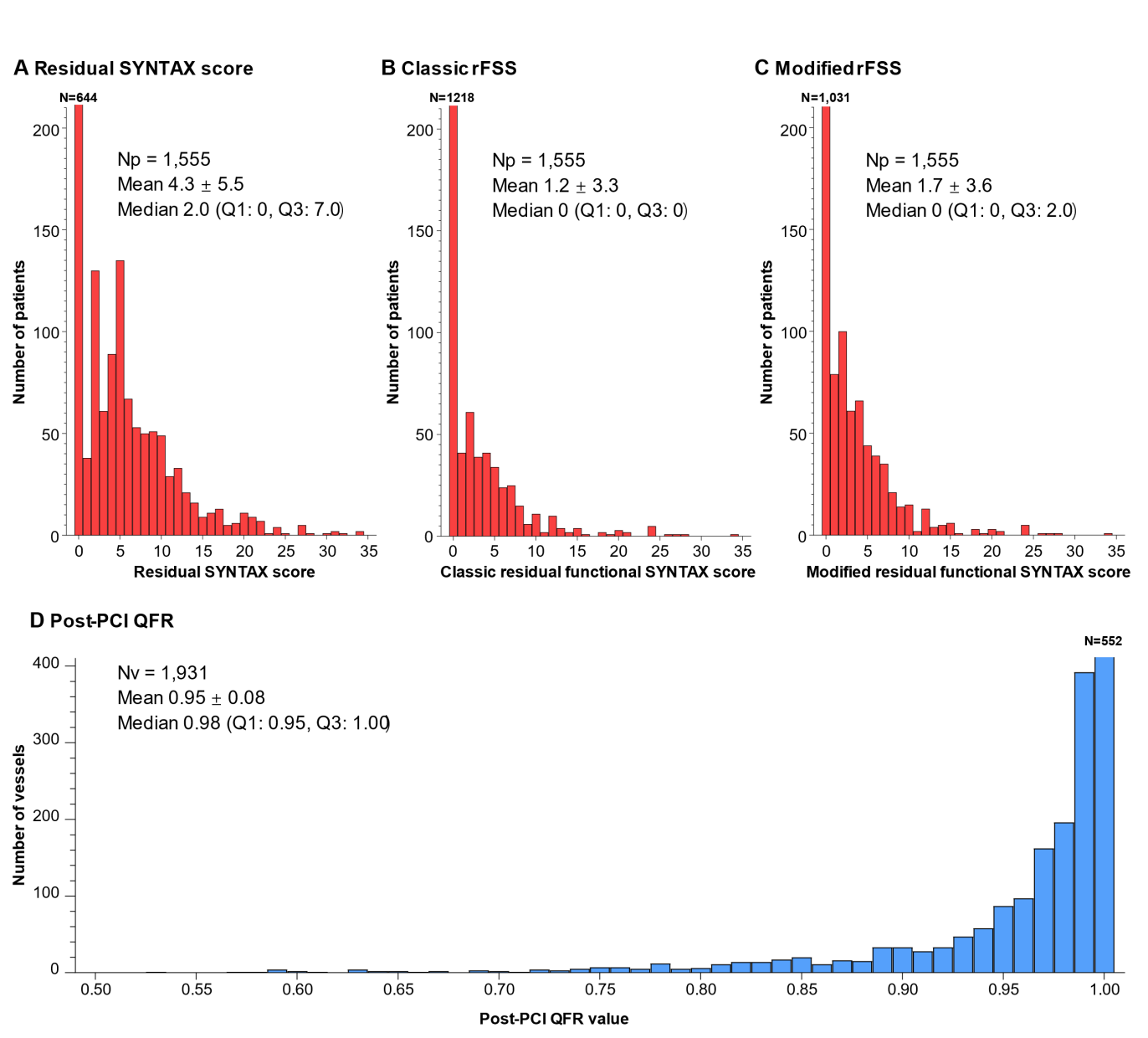
**

**Supplemental Figure 3. Correlations Among Residual SYNTAX Score, Classic Residual Functional SYNTAX Score and Modified Residual Functional SYNTAX Score**

Correlations (A) between anatomic residual SYNTAX score (rSS) and classic residual functional SYNTAX score (c-rFSS), (B) between rSS and modified residual functional SYNTAX score (m-rFSS), and (C) between c-rFSS and m-rFSS are presented.

Abbreviations as in Figures S1 and S2.

**
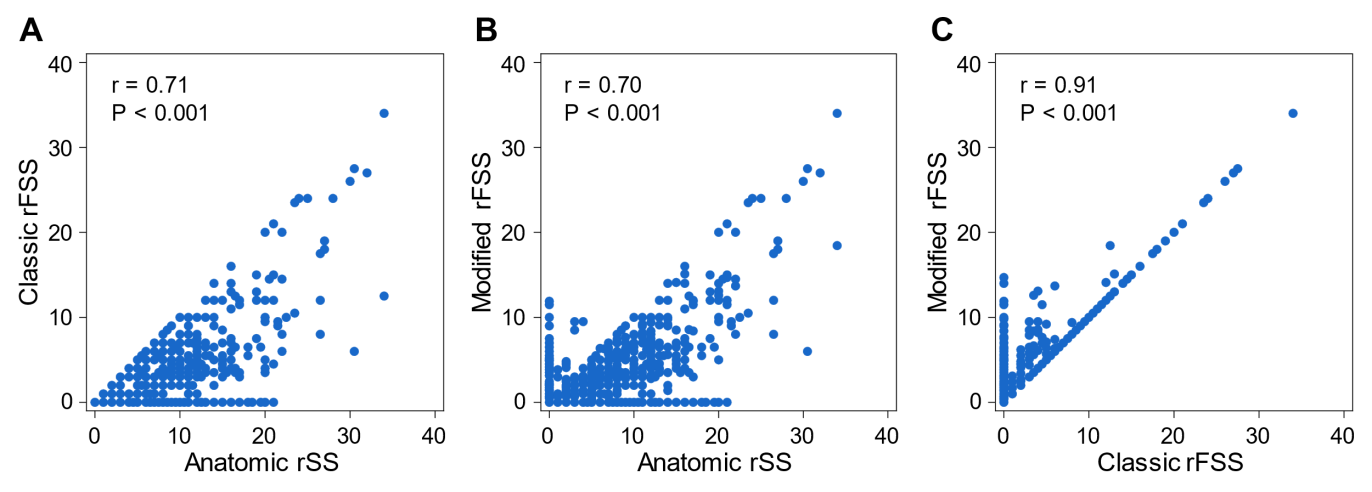
**

**Supplemental Figure 4. Reclassification by Modified Residual Functional SYNTAX Score**

Abbreviations as in Figures S1 and S2.


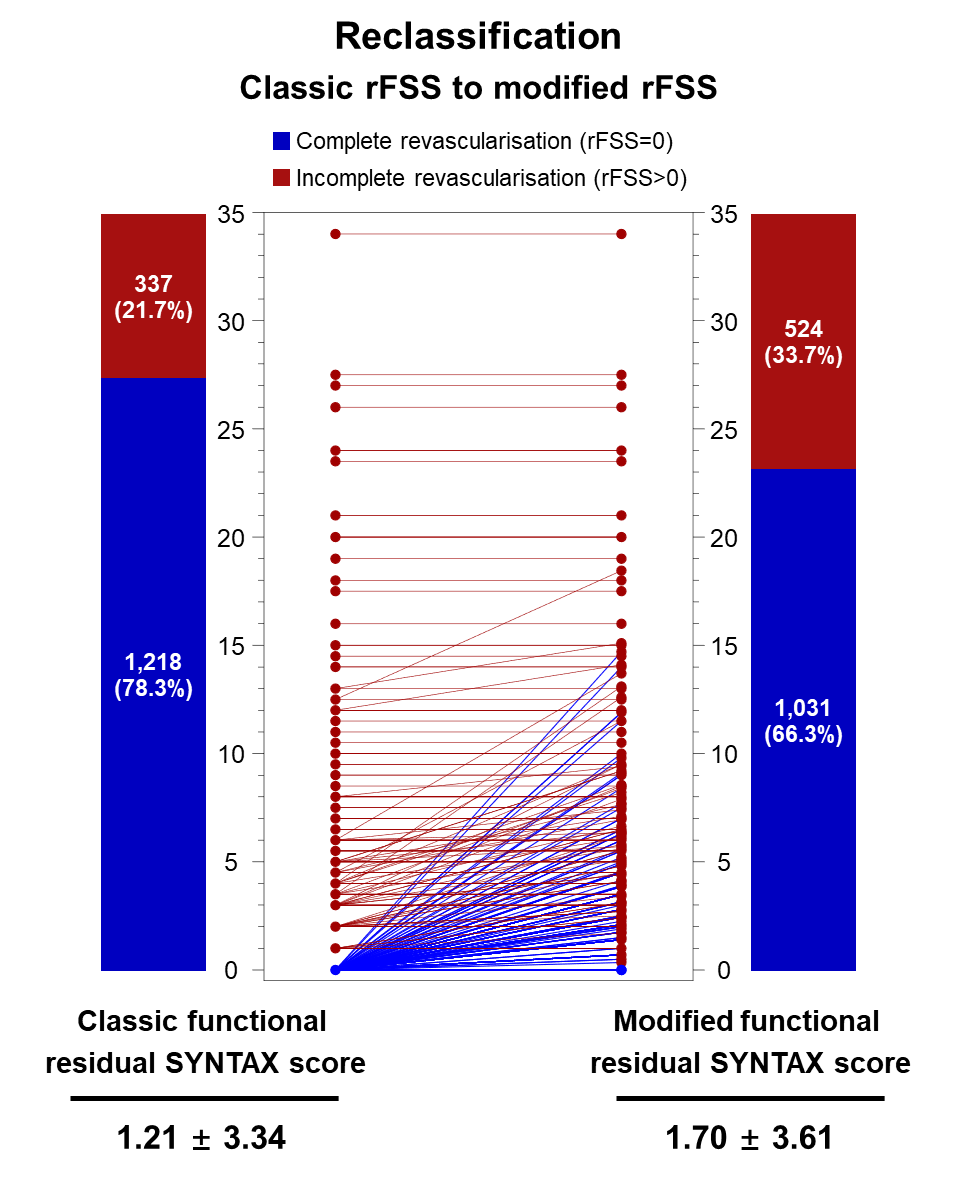


**Supplemental Figure 5. Subgroup Analyses**

Abbreviations as in Figures S1 and S2.

**
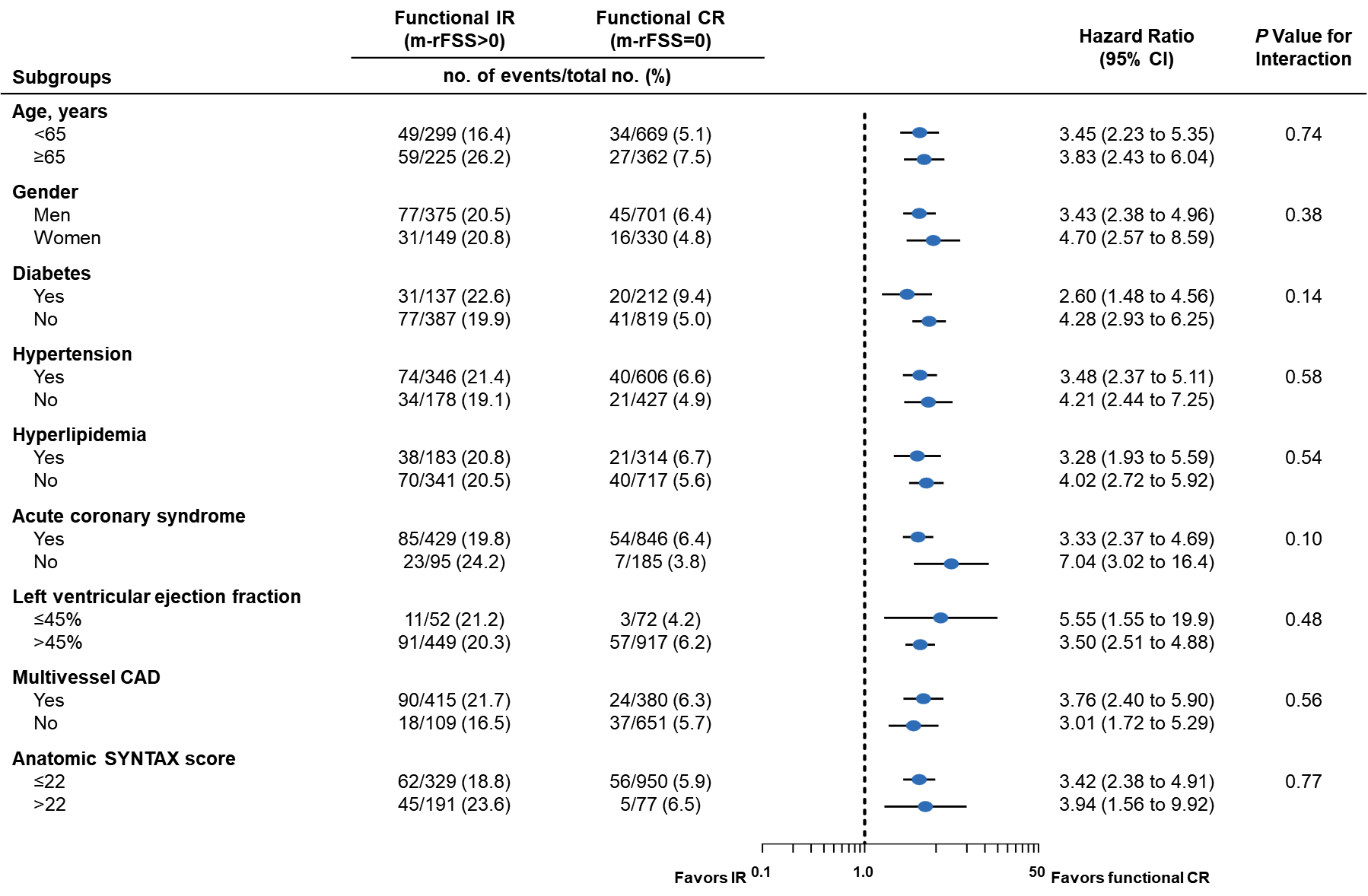
**

**Supplemental Figure 6. Association Between the Estimated risk of Clinical Events and continuous rSS, c-rFSS or m-rFSS**

Estimated risks of 2-year MACE and MACE excluding peri-procedural myocardial infarction according to anatomic residual SNYTAX score (A and D), classic residual functional SNYTAX score (B and E) or modified residual functional SNYTAX score (C and F), respectively.

CI = confidence interval; HR = hazard ratio; MACE = major adverse composite endpoint; MI = myocardial infarction; other abbreviations as in Figures S1 and S2.

**
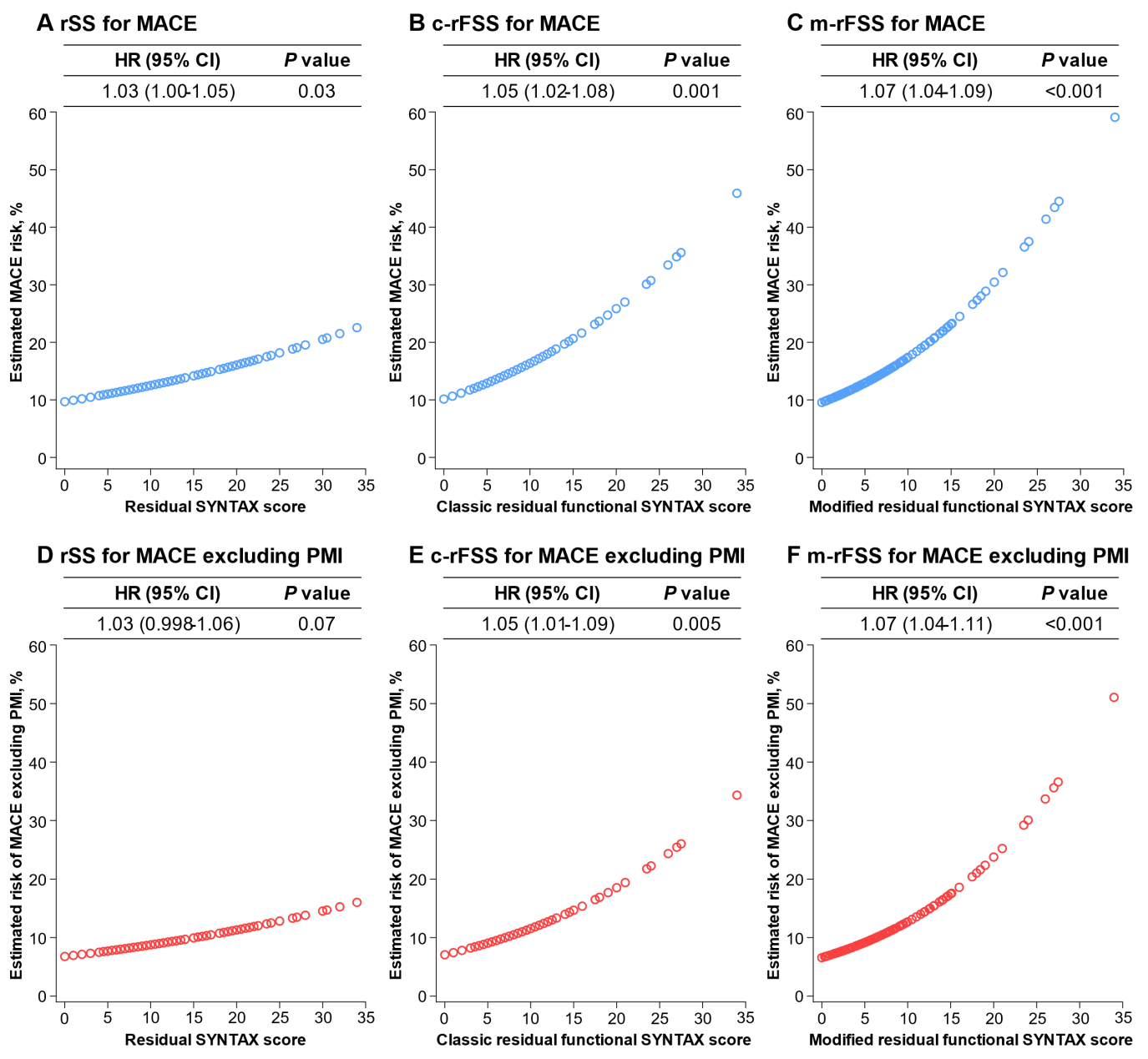
**

**Supplemental Figure 7. Receiver-operating characteristic curve of Anatomic rSS, c-rFSS, or m-rFSS for predicting 2-year Major Adverse Composite Endpoint**

AUC = area under the curve; CI = confidence interval; NRI = net reclassification improvement; IDI = integrated discrimination index; other abbreviations as in Figures S1 and S2.


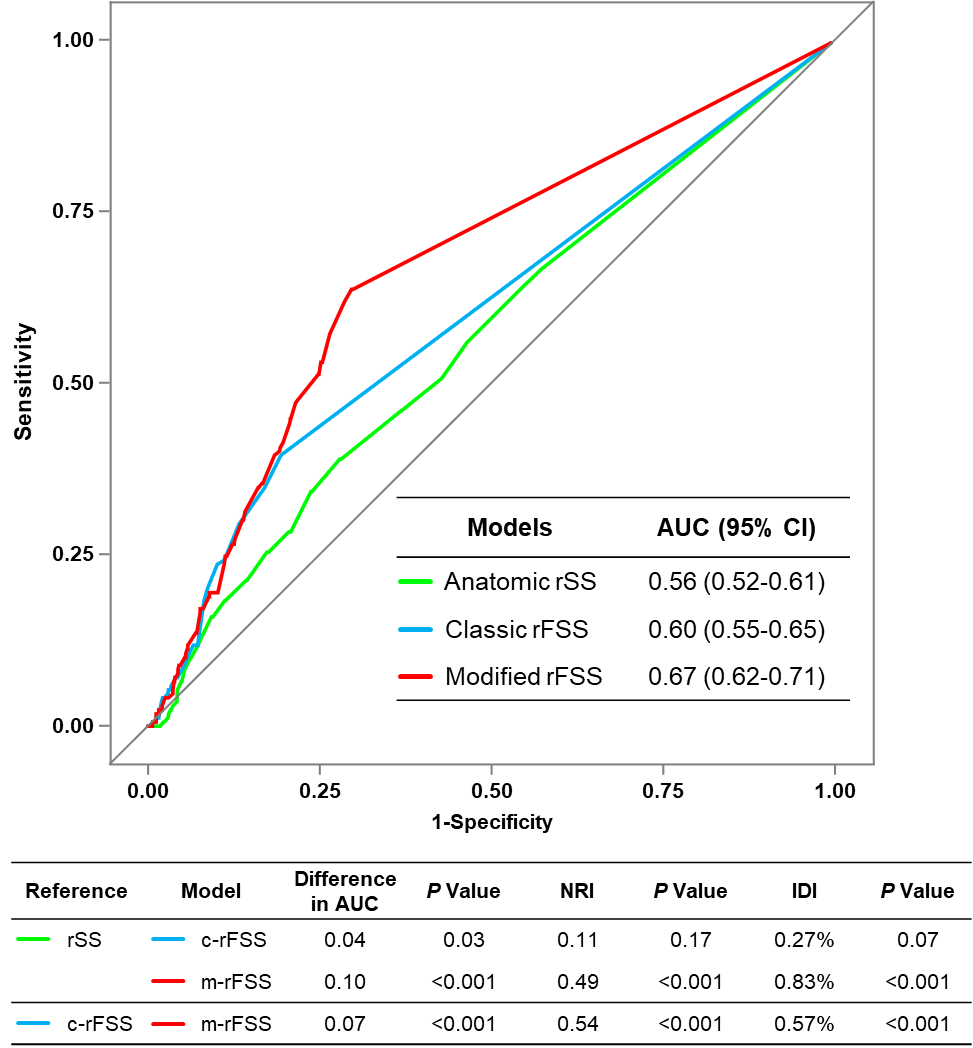


**Supplemental Figure 8. Calibration Plots of Models with rSS, c-rFSS or m-rFSS in Addition to Clinical Risk Factors**

The reference model (model 1) included clinical risk factors only, including age, sex, hypertension, diabetes mellitus, hyperlipidemia, family history of CAD, history of myocardial infarction, and acute coronary syndrome. Model 2 included clinical risk factors plus rSS. Model 3 included clinical risk factors plus c-rFSS. Model 4 included clinical risk factors plus m-rFSS.

abbreviations as in Figures S1 and S2.


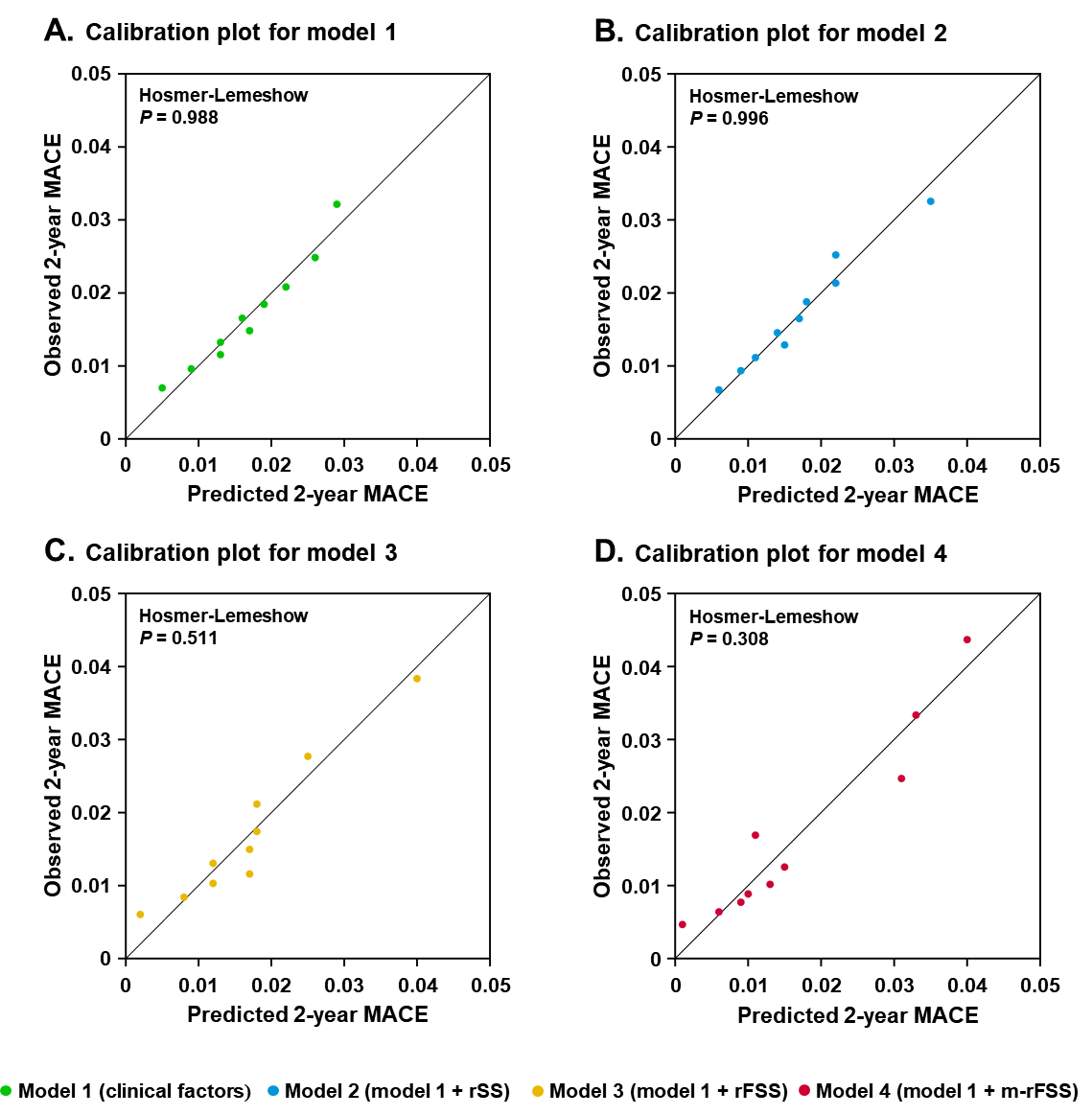

Supplement: Supplementary file 1 — Supporting Information [file ADVS-12-2415961-s001.docx]
